# The contribution of the Golgi and the endoplasmic reticulum to calcium and pH homeostasis in *Toxoplasma gondii*

**DOI:** 10.1016/j.jbc.2025.108372

**Published:** 2025-03-03

**Authors:** Abigail Calixto, Katherine E. Moen, Silvia N.J. Moreno

**Affiliations:** 1 Center for Tropical and Emerging Global Diseases, University of Georgia, Athens, Georgia, USA; 2 Department of Microbiology, University of Georgia, Athens, Georgia, USA; 3 Department of Cellular Biology, University of Georgia, Athens, Georgia, USA

**Keywords:** *Toxoplasma gondii*, calcium signaling, SERCA, pH homeostasis, calcium proton exchanger

## Abstract

The cytosolic Ca^2+^ concentration of all cells is highly regulated demanding the coordinated operation of Ca^2+^ pumps, channels, exchangers, and binding proteins. In the protozoan parasite *Toxoplasma gondii*, calcium homeostasis, essential for signaling, governs critical virulence traits. However, the identity of most molecular players involved in signaling and homeostasis in *T. gondii* is unknown or poorly characterized. In this work, we studied a putative calcium proton exchanger, TgGT1_319550 (TgCAXL1), which belongs to a family of Ca^2+^/proton exchangers that localize to the Golgi apparatus. We localized TgCAXL1 to the Golgi and the endoplasmic reticulum (ER) of *T. gondii* and validated its role as a Ca^2+^/proton exchanger by yeast complementation. Characterization of a knock-out mutant for TgCAXL1 (*Δcaxl*) underscored the role of TgCAXL1 in Ca^2+^ storage by the ER and acidic stores, most likely the Golgi. Most interestingly, TgCAXL1 function is linked to the Ca^2+^ pumping activity of the Sarcoendoplasmic Reticulum Ca^2+^-ATPase (TgSERCA). TgCAXL1 functions in cytosolic pH regulation and recovery from acidic stress. Our data showed for the first time the role of the Golgi in storing and modulating Ca^2+^ signaling in *T. gondii* and the potential link between pH regulation and TgSERCA activity, which is essential for filling intracellular stores with Ca^2+^.

*Toxoplasma gondii* (*T. gondii*) is an Apicomplexan parasite that infects approximately one third of the world's population (1). Most infections with *T. gondii* are asymptomatic but infected immune-deficient patients can develop serious diseases like pneumonia or encephalitis (2). In addition, *T. gondii* infection during pregnancy could cause serious malformations in congenitally infected children (3, 4). The pathogenesis of *T. gondii* is linked to its lytic cycle, which consists of attachment and invasion of host cells, replication within a parasitophorous vacuole, egress from the host cell, and gliding motility to locate and invade a new cell, repeating the cycle (5, 6).

Calcium (Ca^2+^) signaling is universal and impacts almost every aspect of cellular life (7). In *T. gondii*, Ca^2+^ signaling plays a major role throughout the tachyzoite lytic cycle (8, 9, 10, 11, 12). *T. gondii* tachyzoites, like other cells, maintain their cytosolic Ca^2+^ at less than 100 nM (13) as high sustained cytosolic Ca^2+^ is deleterious for all cells (14). *T. gondii* stores Ca^2+^ in organelles like the endoplasmic reticulum and acidic organelles like acidocalcisomes or the plant-like vacuolar compartment (PLVAC) (15, 16). In *T. gondii*, the endoplasmic reticulum (ER) serves as a major Ca^2+^ store, and pumping of Ca^2+^ occurs by a thapsigargin-sensitive Sarcoendoplasmic Reticulum Ca^2+^ ATPase (TgSERCA) (17, 18). Mobilization of Ca^2+^ into the acidocalcisome occurs by the Ca^2+^ ATPase TgA1, which is also expressed at the plasma membrane (19). The PLVAC has also been shown to store Ca^2+^ and likely exports it *via* a Ca^2+^/H^+^ exchanger (15).

Ca^2+^/H^+^ exchangers are essential elements of the Ca^2+^ signaling toolkit of all cells and function to transport Ca^2+^ across membranes in exchange for protons (20). The CAX (CAtion eXchanger) family is responsible for Ca^2+^ uptake, mainly into acidic stores, although there are reports of plasma membrane-localized transporters (21). Members of the CAX family are found in fungi, plants, protozoans, lower vertebrates, and bacteria, but interestingly, not in mammals (22). The *T. gondii* ortholog, TgCAX, was proposed to localize to the PLVAC, but the results were inconclusive due to its low levels of expression (23).

A family of conserved Ca^2+^ antiporters known as UPF0016 proteins for Unknown Protein Family (UPF) 0016, was first described in mammalian cells (24) and the human ortholog, HsTMEM165, was localized to the Golgi. Mutations in the *HsTMEM165* gene have been linked to congenital disorders of glycosylation. The UPF0016 proteins form a conserved family of Ca^2+^ transporters that contain six transmembrane domains and two unique consensus motifs (EXGD-K/R-T/S) (25). The *T. gondii* genome supports the presence of an ortholog of the mammalian gene, *TgGT1_319550* which we termed TgCAXL1 in this publication. The yeast ortholog, ScGDT1, was characterized and localized to Golgi bodies (26). Both HsTMEM165 and ScGDT1 possessed Ca^2+^ transport activity. Whole-cell patch clamp experiments of HeLa cells expressing HsTMEM165 cells displayed ionic currents in response to Ca^2+^ (26). Experiments with the chemical indicator, FURA-2, in *Lactococcus lactis* expressing HsTMEM165 or ScGDT1, demonstrated the ability of the proteins to mobilize Ca^2+^ across the plasma membrane (27, 28). HsTMEM165 and ScGDT1 are important for tolerating high concentrations of Ca^2+^, supporting a role in Ca^2+^ homeostasis (26). Ca^2+^ transport mediated by GDT1 was dependent on pH, and lysosomes of HeLa cells deficient in TMEM165 were more acidic than wildtype (26, 27). Ca^2+^-dependent phenotypes are exacerbated in double mutants of ScGDT1 and the Golgi-specific Ca^2+^-ATPase ScPmr1, suggesting an interaction between these two proteins (26, 27, 29).

In this work, we characterized the function of *T. gondii* Ca^2+^/proton exchanger-like protein 1 (TgCAXL1) (TgGT1_319550) in Ca^2+^ signaling, pH homeostasis and its modulation of TgSERCA Ca^2+^-pumping activity. Our findings underscore the interplay between Ca^2+^ and pH, highlighting the role of Ca^2+^/H^+^ exchangers in Ca^2+^ homeostasis. In addition, our work highlights the Golgi as part of the Ca^2+^ signaling toolkit of *T. gondii*.

## Results

### CAXL1 is part of a novel family of Ca^2+^ antiporters

**Figure 1:**
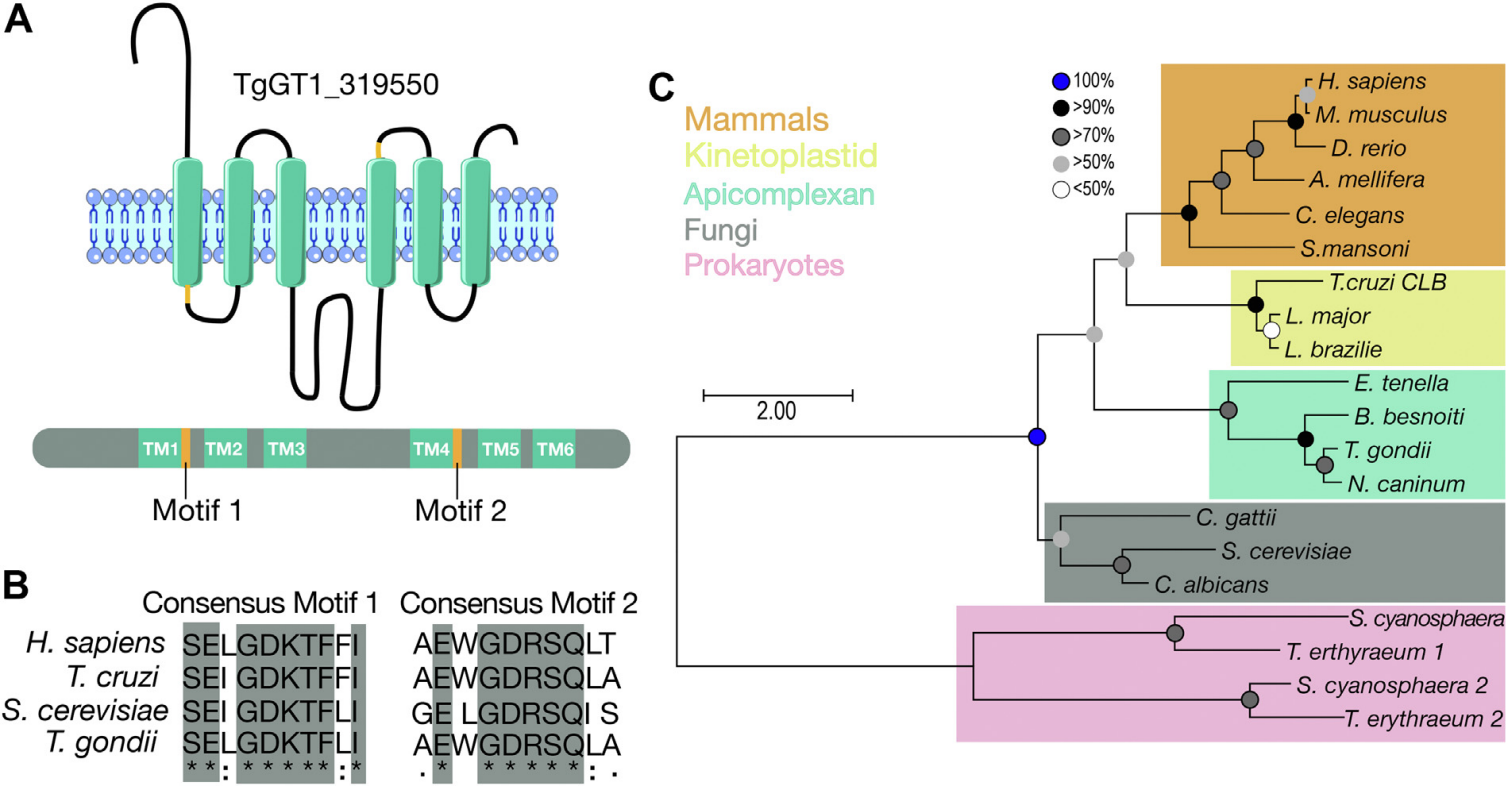
**Phylogenetic placing of the *T. gondii TgCAXL1* gene**. *A*, protter prediction and gene annotation of *TgGT1_319550*. TM = transmembrane domain. *B*, multiple sequence alignment of conserved motifs in TgGT1_319550 orthologs using ClustalW multiple sequence alignment. *Gray* indicates fully conserved amino acids. *C*, phylogenetic analysis of UPF0016 family orthologs. Accession numbers and full-length names for each organism are listed in Table S1. The scale indicates branch length as the number of substitutions per site.

The *T. gondii* ortholog gene of the UPF0016, TgGT1_319550 (TgCAXL1) is predicted to possess six transmembrane domains according to Protter (30) and its sequence contains the two characteristic consensus motifs (EXGD-K/R-T/S) (Fig. 1, *A* and *B*). We generated a maximum likelihood tree to look at the relationship of TgCAXL1 with other orthologs and saw that there was a clear divergence between prokaryotic and eukaryotic orthologs (Fig. 1*C*). The evolutionary relationships amongst representative metazoans and fungi members of the UPF0016 subfamily have been previously analyzed by Demaegd *et al.* (25). Our analysis showed that metazoans cluster together as well as members of the fungi kingdom, similar to the observations of Demaegd *et al.* (25). We included UPF0016 members from two protozoan groups: kinetoplastids and coccidian. *T. gondii* belongs to the coccidia subclass, the largest group of apicomplexan protozoa. Kinetoplastids are flagellated protozoan parasites characterized by the presence of the kinetoplast, an organelle within the mitochondrion that contains its own DNA (31). The kinetoplastid members clustered separately from coccidian members, indicating that they must possess specific functions associated with their parasitic lifestyle. The close evolutionary relationship amongst coccidians implies functional similarities within this group of protists. These data indicate that TgCAXL1 is phylogenetically divergent from other eukaryotes orthologs, but it is conserved within apicomplexans.

### CAXL1 localizes to the endoplasmic reticulum and Golgi apparatus

**Figure 2:**
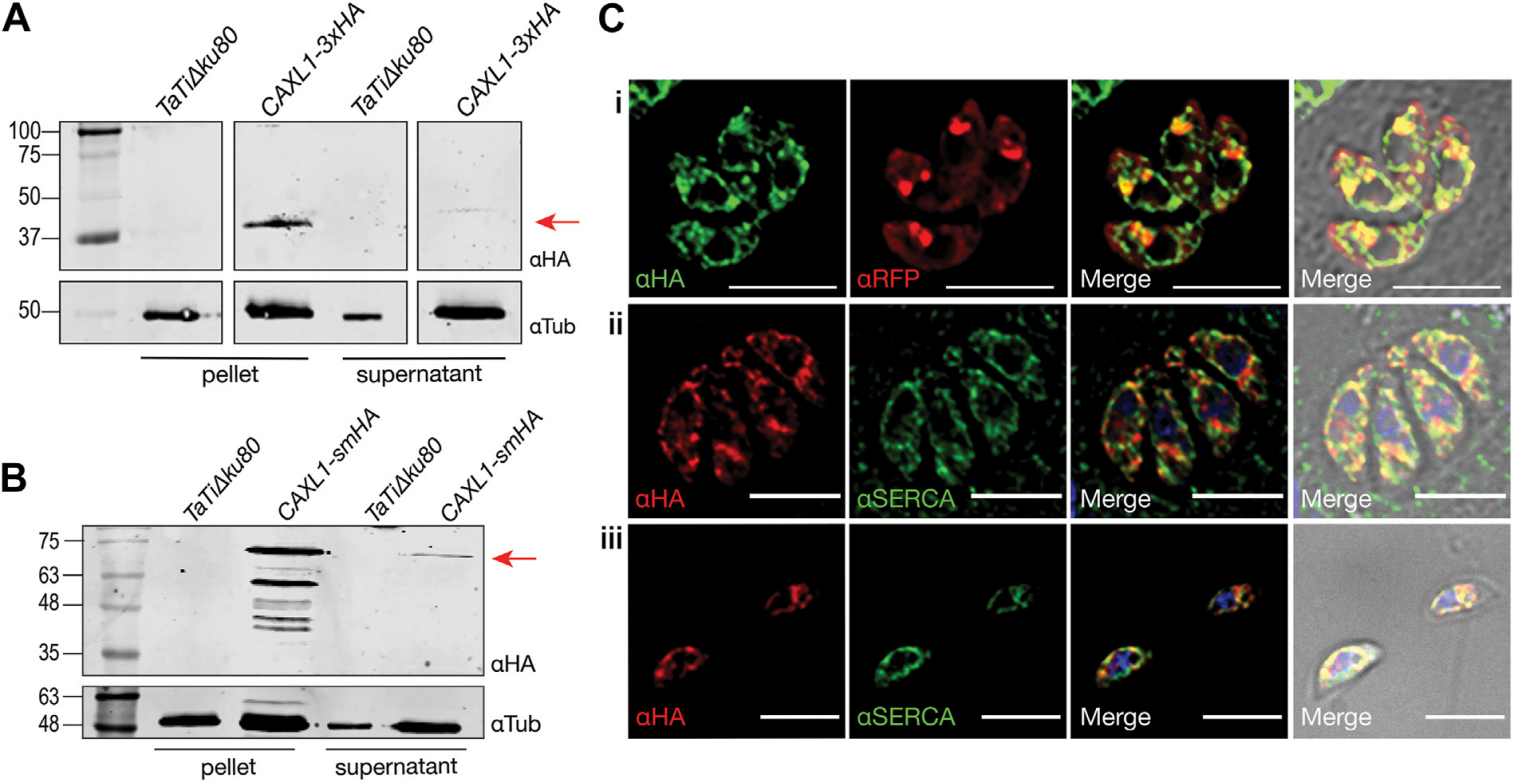
**TgCAXL1 localizes to the Golgi apparatus and the endoplasmic reticulum.***A*, Western blot of CAXL1-3xHA lysate after membrane protein enrichment with anti-HA antibody (αHA) and anti-tubulin (αTub) as a loading control. Arrow points to the expected band size, 38 kDa. *B*, Western blot of a *T. gondii* lysate from the TgCAXL1-smHA following membrane protein enrichment with anti-HA antibody (αHA) and anti-tubulin (αTub) as a loading control. The arrow indicates expected band size, 76 kDa. *C*, Immunofluorescence assays (IFAs) of *T. gondii* parasites using (i) anti-HA antibody and anti-RFP labeling the Golgi marker GRASP55 in intracellular parasites and IFAs using anti-HA antibody and ER marker α-SERCA (ii) in intracellular (Pearson coefficient = 0.6946) and (iii) extracellular parasites (Pearson coefficient = *bottom left*, 0.8974 and *upper right*, 0.9239). The scale bar is 5 μm.

The identification of integral membrane proteins is difficult due to their low level of expression. We endogenously tagged the C-terminus of the *TgCAXL1* gene with a 3-copy hemagglutinin tag (3xHA). We used CRISPR/Cas9 (32, 33) to increase efficiency and the *T. gondii TATiΔKu80* line (34, 35) as in this cell line homologous recombination is favored. Clonal cell lines of TgCAXL1-3xHA were first analyzed *via* PCR and western blots (Fig. 2*A*). We prepared an enriched membrane fraction following an established protocol after lysing parasites by several freeze-thaw cycles. Western blots revealed a band of the expected size, ∼38 kDa (35 kDa for TgCAXL1 plus the 3xHA tag) in the TgCAXL1-3xHA pellet, which was absent in the negative control, supporting correct tagging of TgCAXL1 (Fig. 2*A*).

To determine the localization of TgCAXL1, we performed an immunofluorescence analysis of the TgCAXL1-3xHA mutant with anti-HA antibody. Previous studies showed that TgCAXL1 orthologs localize to the Golgi apparatus, so we tested the Golgi marker GRASP55 (36) and saw colocalization with TgCAXL1 in intracellular parasites transiently expressing GRASP55-RFP (Fig. S1*A*). We noticed an additional signal surrounding the nucleus and considered potential localization to the ER. To test this, we used the ER marker SERCA (αSERCA) and saw that it co-localized with anti-HA (Fig. S1*B*) indicating localization of TgCAXL1 to the ER and the Golgi apparatus, also observed in extracellular tachyzoites (Fig. S1*C*). However, considering the low level of expression of membrane proteins, we tested a previously described strategy of inserting a 10-copy hemagglutinin tag (smHA) (37) at the C-terminus of the *TgCAXL1* gene (Fig. 2*B*). We isolated a clonal cell line, prepared a membrane fraction, and performed Western blots of the pellet and supernatant. In the pellet fraction of TgCAXL1-smHA parasites, we detected a band of the expected size (∼76 kDa), corresponding to TgCAXL1 (35 kDa) plus the smHA tag. The signal was absent in the *TatiΔku80* parental line supporting the correct tagging of TgCAXL1. Some signal was detected in the supernatant that could be contamination from the pellet. Several bands of smaller sizes were detected in the pellet due to potential protein degradation (Fig. 2*B*). Immunofluorescence assays of the TgCAXL1-smHA clone using anti-HA antibody verified co-localization with the ER marker SERCA (αSERCA) in intracellular and extracellular parasites, as well as co-localization in intracellular parasites with GRASP55 (36) in the Golgi apparatus (Fig. 2*C*). TgCAXL1-3xHA immunofluorescence studies confirmed that the smHA tag does not affect the trafficking or localization of the TgCAXL1. Co-localization of TgCAXL1-smHA with the vacuolar H^+^-pyrophosphatase (αVP1) or the Zinc transporter (acidocalcisomes and PLVAC) was not supported by Pearson coefficient analysis, although minimal overlap was observed (Fig. S1, *D* and *E*).

In summary, we successfully tagged TgCAXL1 with an endogenous epitope tag and determined its localization to the Golgi apparatus and the ER.

### TgCAXL1 role in the *T. gondii* lytic cycle

**Figure 3:**
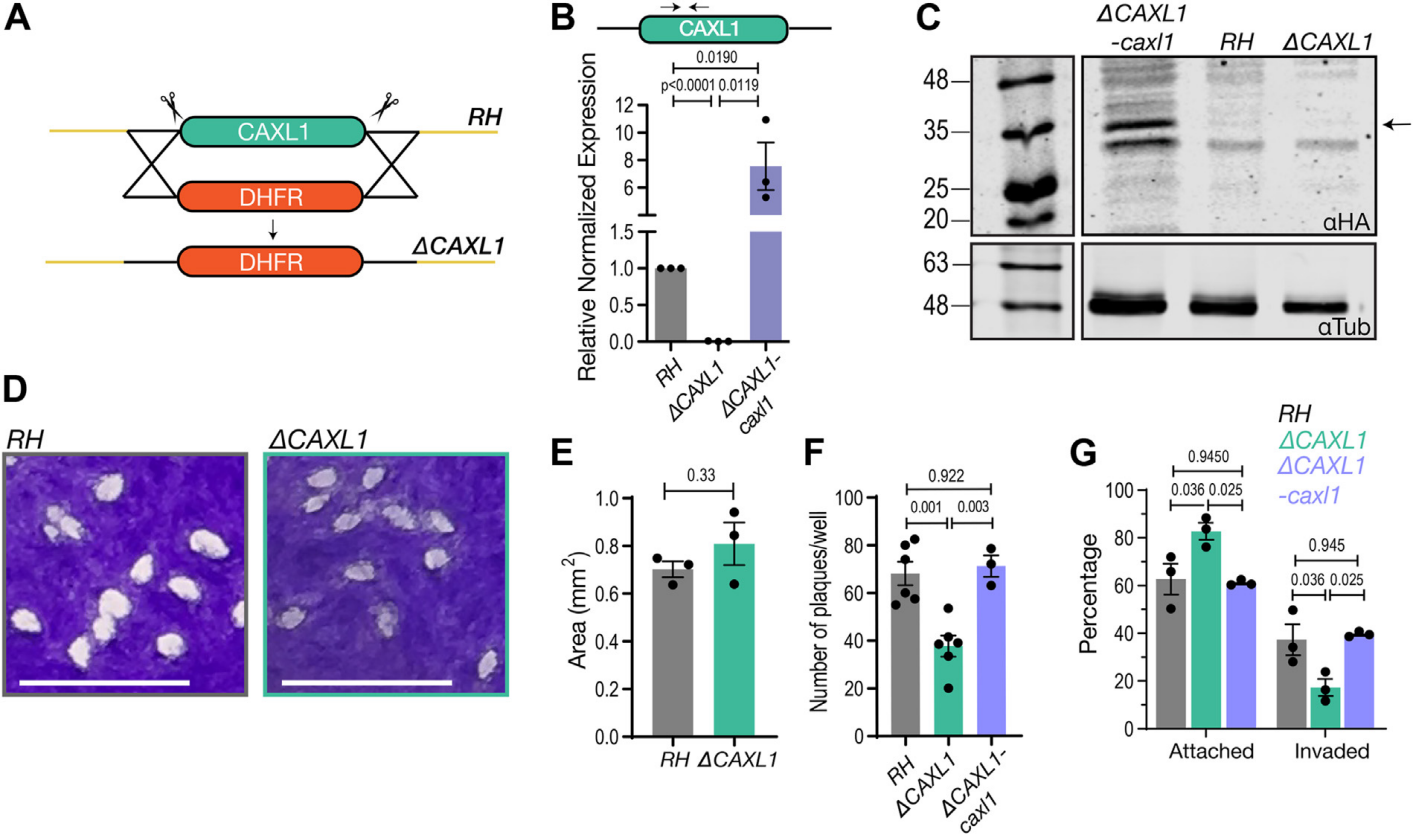
***TgCAXL1* is important for *T. gondii* invasion of host cells.***A*, repair template used to create a KO in the RH wildtype strain, consisting of a dihydrofolate reductase (DHFR) marker flanked with a 1 kilobase homologous region to the 5′ and 3′ UTRs of *TgCAXL1*. Scissors indicate the location of the protospacer motif designed to guide the Cas9 to induce double-stranded breaks. *B*, quantification of gene expression by quantitative PCR. Relative expression is measured in relation to RH, set to 1. Expression values are from three independent biological replicates done in triplicate. Arrows indicate the location of primers within *TgCAXL1*. *C*, Western blot with total lysates from *ΔCAXL1-caxl1*-3xHA (*ΔCAXL1-caxl1*), RH, and *ΔCAXL1* cells with anti-HA (αHA) antibody and anti-tubulin (αTub) as loading controls. Expected band size for CAXL1-3xHA, 39 kDa, is marked with an *arrow*. *D*, Plaque assays after infecting confluent hTert cells with 200 RH or *ΔCAXL1* parasites. The host cell monolayer was fixed with 70% ethanol and stained with crystal violet. White “spots” in the stained host cell monolayer indicate host cell lysis. Scale bar = 5 mm. *E*, the average plaque area from (*D*) quantified using ImageJ. *F*, plaquing efficiency assay. hTERT host cells were allowed to reach full confluency (7 days) and then infected with 1000 parasites for 30 min. The host cell monolayer was washed, and parasites were allowed to grow for an additional 4 days. Wells were fixed with 70% ethanol and stained with crystal *violet* and the number of plaques quantified. *G*, 2 × 10^7^ parasites were allowed to infect HFF cells for 5 min and then fixed with 3% paraformaldehyde. Rabbit anti-SAG1 was used to identify extracellular parasites. Parasites and HFF cells were then permeabilized with 1% Triton X-100 and Mouse anti-SAG1 antibody was used for identification of intracellular parasites. Alexa Fluor 546 Goat anti-Rabbit secondary antibody was used to stain extracellular parasites (*red*) and Alexa Fluor 488 Goat anti-Mouse was used for intracellular parasites (*green*). The graph shows the percentage of parasites invaded and attached from the *red-green* assay. At least 100 parasites were analyzed for each cell line. *B*, *D*– *F*, mean values ± SEM are from three independent biological replicates. *B* and *E* were analyzed using Student *t* test. *F* and *G*, was analyzed using one-way ANOVA.

To investigate the biological function of TgCAXL1 in the parasites, we generated knockout mutants for the *TgCAXL1* gene (Fig. S2, *A* and *B*). *TgCAXL1* has a predicted dispensable CRISPR phenotypic score (38) so we decided to perform a clean deletion of the gene in the RH type I strain using a CRISPR/Cas9 approach. A DHFR drug cassette was constructed flanked with 1 kb homology arms corresponding to the 5′ and 3′ UTR of *TgCAXL1* (Fig. 3*A*). Additionally, two sgRNAs were designed, one specific to the 5′ end and one specific to the 3′ end to increase the likelihood of a clean deletion. Both sgRNAs were transfected simultaneously with the drug cassette and drug-resistant clones were selected. Relative expression of *TgCAXL1* was measured in one of the clones (*ΔCAXL1*) (Fig. 3*B*). The data showed no transcription of *TgCAXL1* compared to wildtype RH, confirming that we had successfully generated a *TgCAXL1* knockout mutant (*ΔCAXL1*) (Fig. 3*B*). To complement the *ΔCAXL1* mutant, we transfected the *ΔCAXL1* clone with the cDNA of *TgCAXL1* fused to a 3xHA at the C-terminus *via* random integration and used chloramphenicol for selection (Fig. S2, *C* and *D*). We selected a clonal population and validated the complemented mutant *(ΔCAXL1-caxl1*) by western blots using an HA antibody as well as with qRT-PCR and observed restoration of TgCAXL1 transcript levels (Fig. 3, *B* and *C*). Immunofluorescence analysis of the *ΔCAXL1-caxl1* mutant confirmed both the expression of TgCAXL1 and its co-localization with SERCA and GRASP55 (Fig. S2*E*). To conclude, we successfully generated both *ΔCAXL1* and *ΔCAXL1-caxl1* cell lines for further biochemical and cellular characterization of TgCAXL1.

We evaluated first the role of TgCAXL1 in tachyzoite growth with plaque assays, in which parasites engage in repetitive cycles of invasion, replication, and egress causing host cell lysis and formation of plaques observed as white spots by staining with crystal violet. Plaque sizes compared between *ΔCAXL1* and RH showed no significant difference (Fig. 3, *D* and *E*). In addition, *ΔCAXL1* and RH were transfected with the red fluorescent protein, tdTomato (39). Parasites were enriched for tdTomato and the mutant clones *ΔCAXL1-RFP* and RH-RFP were isolated. The growth of the red parasites can be visualized and quantified by measuring fluorescence. No difference in the growth kinetics of *ΔCAXL1-RFP* compared to RH-RFP was observed (Fig. S3*A*), and neither replication nor egress was affected by the deletion of *TgCAXL1* (Fig. S3, *B* and *C*). Interestingly, when we characterized the invasion phenotype of the mutant by performing plaquing efficiency assays (Fig. 3*F*) in which parasites are only allowed 30 min of contact with host cells and plaques are quantified 4 days after the initial infection, we observed a significantly reduced number of plaques formed by the *ΔCAXL1* mutant. Further support for the invasion and/or attachment phenotype, was provided with red-green assays, an established protocol to evaluate *T. gondii* invasion (40) (Fig. 3*G*). Approximately 50% less of *ΔCAXL1* parasites were able to invade host cells which correlated with a similar increase in attachment (Fig. 3*G*). Normal invasion and attachment were restored in the complemented mutant *ΔCAXL1-caxl*. In summary TgCAXL1 appears to be important for invasion of host cells.

### Functional analysis of TgCAXL

**Figure 4:**
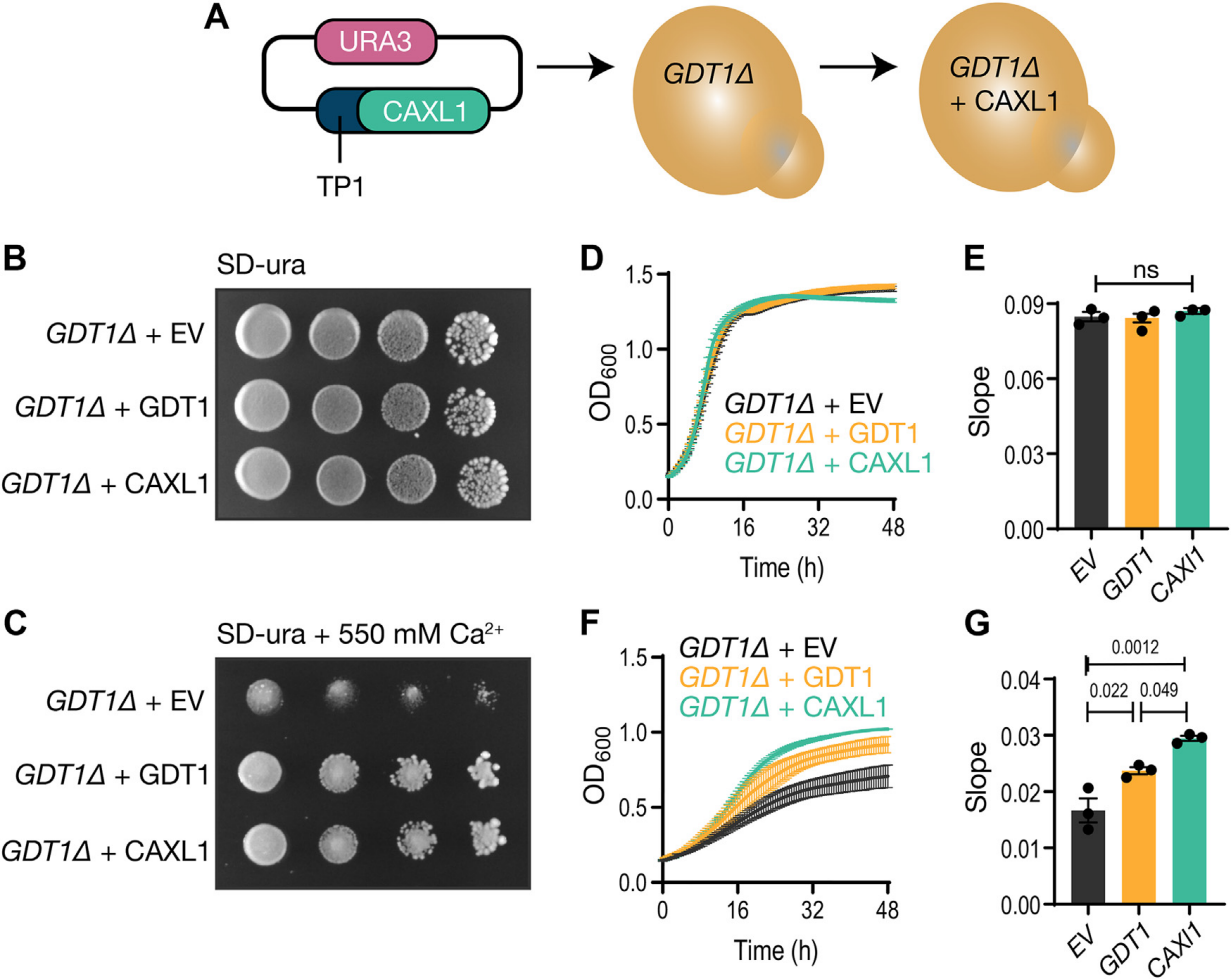
**Expression of *TgCAXL1* protects yeast against high calcium levels.***A*, schematic of *TgCAXL1* complementation of the yeast strain *GDT1Δ. B*, plate growth assay of *GDT1Δ* complemented with the empty vector (EV), or with *GDT1* or *TgCAXL1* constructs. All strains were grown overnight, adjusted to a final OD of 1, and serially diluted at 1:10, 1:100 and 1:1000. 10 μl of each dilution was plated on synthetic defined medium without uracil (SD-ura). *C*, similar conditions to the ones shown in *B*, all strains were precultured to an OD600 of 1 and plated on SD-ura plate supplemented with 550 mM CaCl_2_ and allowed to grow at 30 °C. *D*, growth assay in liquid media of *GDT1Δ* complemented with the empty vector (EV), *GDT1* or *TgCAXL1*. Strains were grown overnight and diluted to an OD of 0.1 for the experiment. The graph shows the absorbance OD_600_ in function of time of SD-ura liquid cultures of yeast grown for 48 h. *E*, quantification of the exponential growth (4–16 h) from (*D*) from three independent biological experiments. *F*, similar experiment to (*D*) except, liquid growth medium is supplemented with 550 mM CaCl_2_. *G*, quantification of the slope of exponential growth phases (8–32 h) from the experiment shown in (*F*) from three independent biological experiments. For (*D* and *F*), graphs represent three independent biological replicates done in triplicate and error bars represent mean ± SEM. For (*E* and *G*), values indicate mean ± SEM and analysis was done using one-way ANOVA. SD, synthetic defined medium; TP1, triose phosphate isomerase 1 promoter.

To study the function of TgCAXL1 we utilized the yeast mutant *GDT1Δ*. In this mutant, the deletion of *GDT1*, the CAXL ortholog, did not affect growth under homeostatic conditions (26). However, when exposed to high Ca^2+^ concentrations (550 mM) the *GDT1Δ* was not able to grow. This result supported a role for GDT1 in regulating Ca^2+^ homeostasis under extreme Ca^2+^ conditions. Taking advantage of the Ca^2+^ sensitivity of the *GDT1Δ* mutant, we tested whether complementation with the *TgCAXL1* gene would restore its growth under extreme Ca^2+^ conditions. To this end, we cloned the *TgCAXL1* gene in a yeast expression plasmid containing the URA3 metabolic marker which favors yeast growth without uracil URA3. We fused the *TgCAXL1* gene with the first 69 bps of the *GDT1* reading frame, which represents the ScGDT1 signal peptide for translocation to Golgi bodies (Fig. 4*A*). We successfully obtained *GDT1Δ*+*TgCAXL1* transformants in this selective media.

Plate growth assays showed no growth defect of *GDT1Δ* (*GDT1Δ* + empty vector (EV)) (26) under homeostatic conditions (CSM-ura) (Fig. 4*B*). However, under high Ca^2+^ concentration, 550 mM Ca^2+^ (CSM-ura + 550 mM Ca^2+^), the growth of *GDT1Δ* + EV was repressed (Fig. 4*C*). Expression of the ScGDT1 restored growth under high Ca^2+^ concentrations. Most interestingly, expression of the *TgCAXL1* gene restored the retarded growth of the *GDT1Δ* mutant under high Ca^2+^ (Fig. 4*C*). This result was further supported by growing the same mutants under identical conditions in liquid media. We evaluated growth for a period of 48 h. The growth of *GDT1Δ* was normal in CSM-ura liquid media (Fig. 4, *D* and *E*), but was repressed in high Ca^2+^ concentrations (550 mM) (Fig. 4, *F* and *G*, *black lane and bar*). This growth defect was rescued during the exponential growth phase by the expression of both the ScGDT1 and TgCAXL1 (Fig. 4*G*). Quantification of growth in liquid media showed increased growth of both the *CAXL1* and native *GDT1* complemented strains (Fig. 4*G*), although the difference between the two complemented strains was barely significant. This could be due to a difference in the level of expression of both genes, or it may suggest that TgCAXL is more efficient at transporting Calcium than GDT1, which also transports manganese, necessary for N-glycosylation in the Golgi (41). These results supported the biochemical function of TgCAXL1 as a Ca^2+^ proton exchanger as it protected yeast from the toxicity of high Ca^2+^ concentrations.

### The role of TgCAXL1 in intracellular Ca^2+^ fluctuations

**Figure 5:**
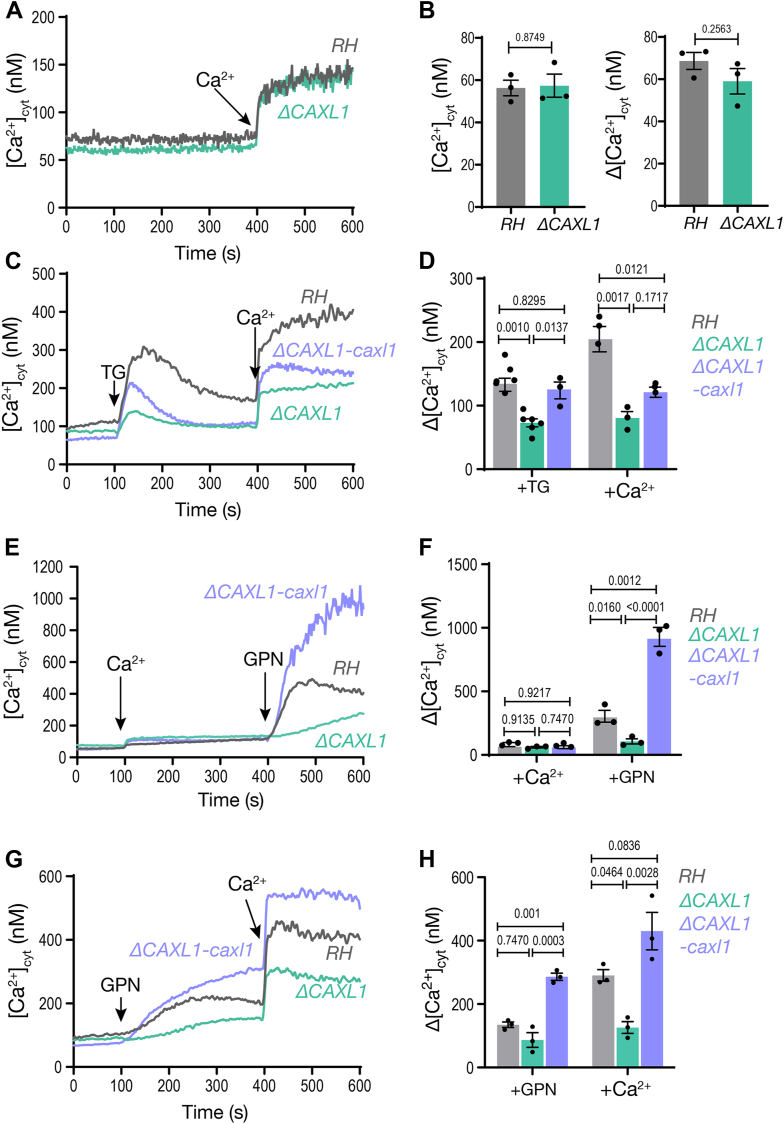
**The role of*****TgCAXL1*****in intracellular Ca^2+^ stores**. *A*, Tachyzoites of the parental RH strain and the Δ*CAXL1* mutant loaded with Fura-2 were analyzed for Ca^2+^ entry. Addition of 1.8 mM Ca^2+^ at 400 s to *T. gondii* tachyzoites in suspension in a Ringer buffer with 100 μM EGTA. The *gray trace* shows the response of the control RH parasites, and the *light green* shows the response of the Δ*CAXL1* mutant. *B*, *Left:* Quantification of the resting cytosolic Ca^2+^ and *Right*: Quantification of the Ca^2+^ peak after addition of 1.8 mM of Ca^2+^. Each dot represents an independent biological experiment. *C*, similar experimental conditions to the ones in *A*. Cytosolic Ca^2+^ increase after the addition of 1 μM thapsigargin (TG) at 100 s followed by 1.8 mM Ca^2+^ at 400 s. The *gray* trace shows the response of the control RH parasites, the *light green* shows the response of the Δ*CAXL1* mutant and the *light blue* the response of the complemented mutant Δ*CAXL1-caxl1*. *D*, quantification of the change in cytosolic Ca^2+^ after addition of TG or Ca^2+^. Δ[Ca^2+^]_cyt_ was calculated by measuring the difference between peak Ca^2+^ and the average Ca^2+^ 20 s prior to the addition of Ca^2+^ or TG. *E*, cytosolic Ca^2+^ increase after the addition of 1.8 mM Ca^2+^ at 100 s followed by 40 μM GPN at 400 s. Similar experimental conditions to the ones in *A*. *F*, quantification of the change in cytosolic Ca^2+^ after addition of Ca^2+^ or GPN. Differences were calculated by measuring the difference between peak Ca^2+^ and the average Ca^2+^ 20 s prior to the addition of Ca^2+^ or GPN. *G*, same experiment as the one presented in (*E*) but additions inverted. GPN was added at 100 s followed by 1.8 mM Ca^2+^ at 400 s. *H*, quantification of the change in cytosolic Ca^2+^ after the addition of GPN or Ca^2+^. Differences were calculated by measuring the difference between peak Ca^2+^ and the average Ca^2+^ 20 s prior to the addition of Ca^2+^ or GPN. Three independent biological replicates were done for all experiments, and each experiment was done in duplicate. For quantification of ΔCa^2+^, mean values ± SEM were analyzed using one-way ANOVA.

To study the role of TgCAXL1 in intracellular Ca^2+^ fluctuations, we loaded control (RH) and *ΔCAXL1* parasites with the Ca^2+^ indicator Fura-2 (Fig. 5*A*). The resting cytosolic Ca^2+^ concentration of the Δ*CAXL1* mutant was ∼60 nM, no different from the resting Ca^2+^ of the parental cell line, RH (11, 13) (Fig. 5*B*, *left bar graph*). Using a similar experimental set-up to previously (11) we tested extracellular Ca^2+^ influx. 1.8 mM Ca^2+^ was added to the extracellular buffer of parasites suspended in a low Ca^2+^ buffer (100 μM EGTA). Under these conditions, an increase in cytosolic Ca^2+^ indicates Ca^2+^ influx. The ΔCa^2+^ due to influx was comparable between *ΔCAXL1* and wildtype cells (RH) (Fig. 5, *A* and *B right graph*). We next tested cytosolic influx from the endoplasmic reticulum by adding thapsigargin (TG) (42), an inhibitor of SERCA resulting in uncompensated ER Ca^2+^ efflux into the cytosol. Interestingly, cytosolic Ca^2+^ efflux from the ER was significantly reduced in the *ΔCAXL1* mutant (Fig. 5, *C* and *D*). We previously showed that elevated cytosolic Ca^2+^ modulates/stimulates plasma membrane Ca^2+^ influx (Ca^2+^ activated Ca^2+^ entry or CACE) (11, 43). We next tested if CACE was affected in the *ΔCAXL1* mutant. For this, we first added TG followed by extracellular Ca^2+^. Because TG elevates cytosolic calcium, in the parental cells this results in an increased Ca^2+^ influx (Fig. 5*C*, *gray traces*, D, *gray bars*). Under similar experimental conditions, the *ΔCAXL1* mutant showed diminished CACE activity (∼50% lower) which was likely due to the reduced increase of cytosolic Ca^2+^ after adding TG (Fig. 5*C*). To further test Ca^2+^ release from intracellular stores, we exposed the parasites to zaprinast, a phosphodiesterase inhibitor which has been shown to cause an increase in cytosolic Ca^2+^ resulting from increased levels of the cyclic nucleotide, cGMP (44, 45). The precise store that zaprinast targets is not clear, but it was hypothesized that it releases Ca^2+^ from neutral stores, potentially the ER (45, 46). Both *ΔCAXL1* and RH showed comparable responses to zaprinast, suggesting that TgCAXL1 did not affect the PKG signaling pathway and specifically impacted Ca^2+^ leakage from the ER after exposure to TG (Fig. S4, *A* and *B*). This could be because of reduced storage of Ca^2+^ in the ER or because of a possible interaction between CAXL1 and the ER leakage mechanism.

We next investigated if the acidic stores were affected in the *ΔCAXL1* mutant as it has been reported that downregulation of the human TMEM165 impacted lysosomal pH (26). We tested the lysosomotropic agent glycyl-L-phenylalanine-2-naphthylamide (GPN), which mobilizes Ca^2+^ from acidic organelles (15, 47, 48, 49). We first added extracellular Ca^2+^ at 100 s to the suspension to allow replenishment of the stores and added GPN at 400 s to release Ca^2+^ from acidic stores (Fig. 5, *E* and *F*). A cytosolic Ca^2+^ increase was observed in the three cell lines: wildtype, *ΔCAXL1* and *ΔCAXL1-caxl1*. Interestingly, the cytosolic Ca^2+^ increase of the *ΔCAXL1* mutant after adding GPN was significantly reduced (Fig. 5, *E* and *F*) and the effect was restored in the complemented mutant *ΔCAXL1-caxl*. This could be because either the *ΔCAXL1* mutant is unable to take up Ca^2+^ into the acidic stores, or the release of Ca^2+^ by GPN is affected in the *ΔCAXL1* mutant. To further investigate this, we tested whether we would see the same defect in parasites with resting levels of Ca^2+^ and 100 μM EGTA in the buffer. Under these conditions, we observed a difference in the cytosolic Ca^2+^ increase after adding GPN (Fig. 5, *G* and *H*), but it was not significant. We measured the area under the curve after the addition of GPN for each cell line and obtained minimal significance (Fig. S4*C*), which could be because of the low level of stored Ca^2+^ (under the conditions of the experiment) in the acidic compartments resulting in slow-release masking the differences between the control and the mutants. Ca^2+^-stimulated Ca^2+^ entry after GPN was also diminished in the *ΔCAXL1* mutant and was restored to control levels in the *ΔCAXL1-caxl* complemented mutant (Fig. 5, *G* and *H*). We noticed that the *ΔCAXL1-caxl1* mutant, which overexpresses *TgCAXL1*, showed an increased response to GPN. It is possible that the GPN-responding store contains more calcium, possibly due to the higher efficiency of calcium uptake from the cytosol caused by overexpression.

The lack of phenotype in the response to Zaprinast of the *ΔCAXL1* mutant led us to investigate further this response. GPN, which mainly releases Ca^2+^ from acidic stores reduced the Zaprinast response and this was seen with parasites pre-incubated with Calcium or pre-incubated with EGTA (Fig. S4, *D*–*G*). This indicated that Zaprinast may be acting on other stores in addition to the ER explaining why the *ΔCAXL1* mutant showed no difference in the response.

We also used NH_4_Cl and the potassium ionophore, nigericin, which are both known to release calcium from acidic stores, and obtained similar results between the RH and the *ΔCAXL1* mutant when quantifying the amount of Ca^2+^ released by both agents. In addition, the Ca^2+^-stimulated Ca^2+^ entry after NH_4_Cl was not affected in the *ΔCAXL1* mutant (Fig. S5).

In summary, the data on cytosolic Ca^2+^ fluctuations indicates that TgCAXL1 is important for release of Ca^2+^ from the ER and acidic stores, likely the Golgi apparatus. It appears that the acidic stores of the *ΔCAXL1* mutant have lost the capacity or have become less efficient at taking up Ca^2+^ following entry from the extracellular environment. The reduced cytosolic efflux of Ca^2+^ subsequently impacted Ca^2+^ entry.

### Impact of TgCAXL1 in the activity of TgSERCA

**Figure 6:**
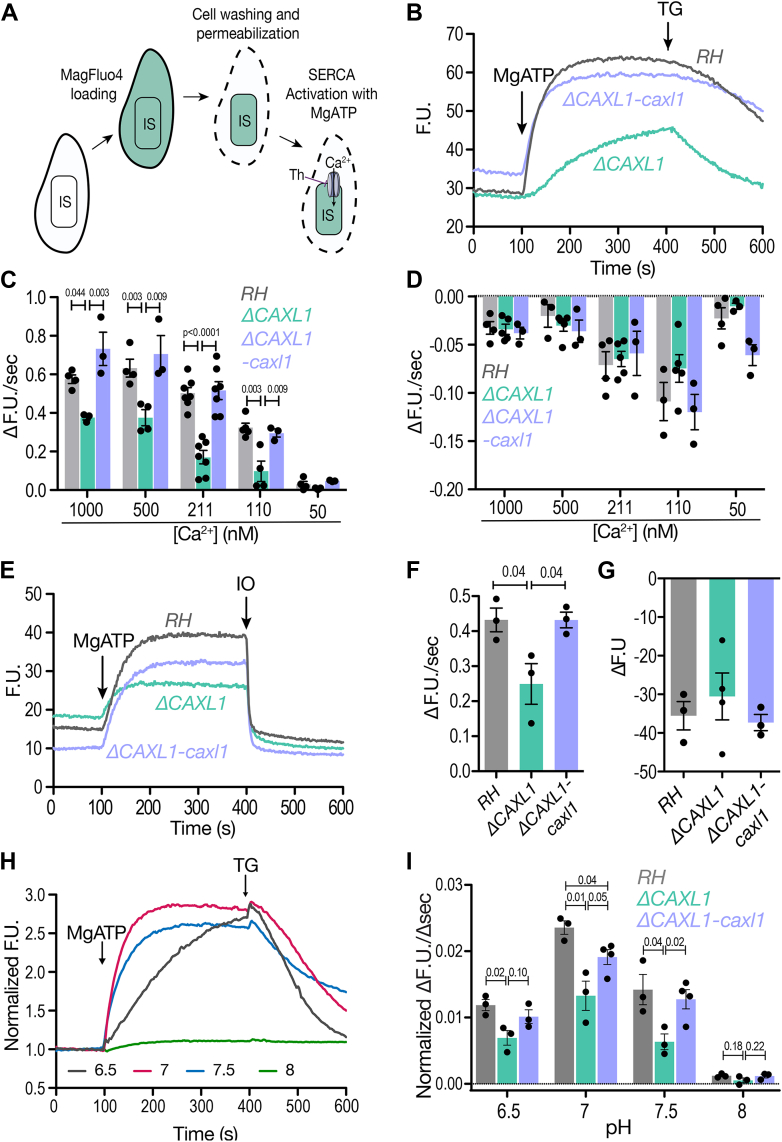
**TgCAXL1 is important for SERCA Ca^2+^ pumping.***A*, model of MagFluo-4-AM loading in parasites. MagFluo-4 disperses throughout the parasite during loading. After permeabilization with digitonin, MagFluo-4 is washed out of the cytosol and retained in the intracellular stores. MagFluo-4 fluorescence will increase when binding calcium and the addition of MgATP will activate TgSERCA. Addition of TG will cause Ca^2+^ release from the ER. *B*, representative tracings of the MagFluo-4 fluorescence. Parasites were resuspended in CLM buffer containing 211 nM free Ca^2+^. 250 μM MgATP was added at 100 s and 1.5 μM thapsigargin (TG) at 400 s. *C*, quantification of the slope after the addition of MgATP at various Ca^2+^ concentrations in the CLM buffer. The slope was calculated between 100 and 140 s. *D*, quantification of the slope after the addition of 1.5 μM TG at various calcium concentrations. The slope was calculated between 400 to 600 s. *E*, representative tracing of the fluorescence from MagFluo-4 loaded tachyzoites after addition of 250 μM MgATP at 100 s and 1 μM ionomycin (IO) at 400 s in CLM buffer + 211 nM Ca^2+^. *F*, quantification of the slope after the addition of MgATP. The slope was calculated from 100 to 140 s. *G*, quantification of the change in fluorescence after addition of 1 μM IO. *H*, representative tracings of the fluorescence from MagFluo-4 in permeabilized RH parasites resuspended in CLM buffers at various pHs + 220 nM free Ca^2+^. 250 μM MgATP was added at 100 s and 1.5 μM TG at 400 s. *I*, quantification of the slope after the addition of MgATP in parasites resuspended in CLM buffers with varying pHs. Slopes were analyzed for RH, *ΔCAXL1*, and *ΔCAXL1-caxl1*. The slope was calculated between 100 and 170 s. For all the experiments the *gray* traces indicate the results obtained from RH, *light green* for *ΔCAXL1* and *light blue* for *ΔCAXL1-caxl1*. Values from (*C*, *D*, *F*, *G* and *I*) indicate mean ± SEM and analyzed using one-way ANOVA. TG = Thapsigargin.

To further investigate the mechanism by which TgCAXL1 may impact the ER Ca^2+^ store and/or efflux we loaded parasites (control, *ΔCAXL1*, and *ΔCAXL1-caxl* mutants) with the low-affinity Ca^2+^ indicator MagFluo4-AM (18, 50). We allowed for a longer loading time to permit the compartamentalization of the dye (18). Loaded parasites, were permeabilized to allow the cytoplasmic indicator to wash off allowing for the MagFluo4 fluorescence to measure organellar Ca^2+^ (Fig. 6*A*). The suspension of parasites was exposed to the TgSERCA substrate, MgATP, which activates the Ca^2+^ pumping activity and so the increase in fluorescence indicates Ca^2+^ uptake into the ER and/or Golgi (as SERCA localizes to both compartments). After reaching the fluorescence plateau, we exposed cells to TG, a SERCA inhibitor, which induced Ca^2+^ release/leakage from the ER, resulting in a decrease in fluorescence (Fig. 6*B*). Comparison of Ca^2+^ pumping activity between RH, *ΔCAXL1*, and *ΔCAXL1-caxl1* in a buffer containing 211 nM Ca^2+^ revealed a significantly reduced rate of Ca^2+^ uptake in the *ΔCAXL1* mutant (Fig. 6*B*). The absence of TgCAXL1 significantly decreased SERCA Ca^2+^ uptake activity at various Ca^2+^ concentrations (Fig. 6*C*), except at 50 nM Ca^2+^, most likely because uptake was very low, accounting for the lack of significant difference. The rate of Ca^2+^ leakage after the addition of TG was not affected (Fig. 6*D*), indicating that TgCAXL1 is regulating Ca^2+^ uptake into the ER. These phenotypes were recovered in the *ΔCAXL1*-*caxl* mutant. We next tested the Ca^2+^ ionophore, ionomycin, which allows the release of Ca^2+^ from neutral stores, including the ER (51). The addition of ionomycin evoked a decrease in the MagFluo4 fluorescence following the filling of the stores by the action of the MgATP-stimulated SERCA, as it allowed Ca^2+^ to be released (Figs. 6, *E*–*G* and S6*A*). The apparent difference in the Ca^2+^ released in response to ionomycin between the wildtype strain and the *ΔCAXL1* mutant was not significant as it was variable. These results confirmed that TgCAXL1 directly impacted the Ca^2+^ pumping activity of TgSERCA.

Pumping of Ca^2+^ by SERCA is also accompanied by H^+^ pumping to counterbalance the positive charges being transported (52). As a consequence, proton transport at the ER membrane may impact SERCA activity. It has been shown that the activity of SERCA can be modulated by varying pHs (53). We investigated this and measured uptake activity at neutral pH 7 in the parental line, RH, and the average rate was 0.024 F U./sec. Interestingly, increasing the pH to 7.5, caused a decrease in the SERCA Ca^2+^ uptake activity to approximately, 0.014 F U./sec, a 42% decrease (Fig. 6, *H* and *I*). Furthermore, activity is almost undetectable at pH 8. Under acidic environments (pH 6.5), the SERCA Ca^2+^ uptake rate was also reduced to 0.012 F.U./s. The SERCA activity of the *ΔCAXL1* mutant at pH 8 was not different from the low activity observed in RH parasites. However, at neutral and acidic pH, it was significantly reduced compared to RH parasites (Fig. 6*I*). TgCAXL1 did not appear to impact the leakage of Ca^2+^ from the ER after the addition of thapsigargin at acidic or alkaline pHs (Fig. S6, *B* and *C*). These data established for the first time, a role for pH in the regulation of the activity of TgSERCA. It is possible that the concentration of H^+^ at the ER membrane may have a direct impact on the Ca^2+^ pumping activity of TgSERCA and the function of TgCAXL would be important for keeping the optimal concentration of H^+^ for activity of the Ca^2+^ pump.

### TgCAXL1 is important to control intracellular pH

**Figure 7:**
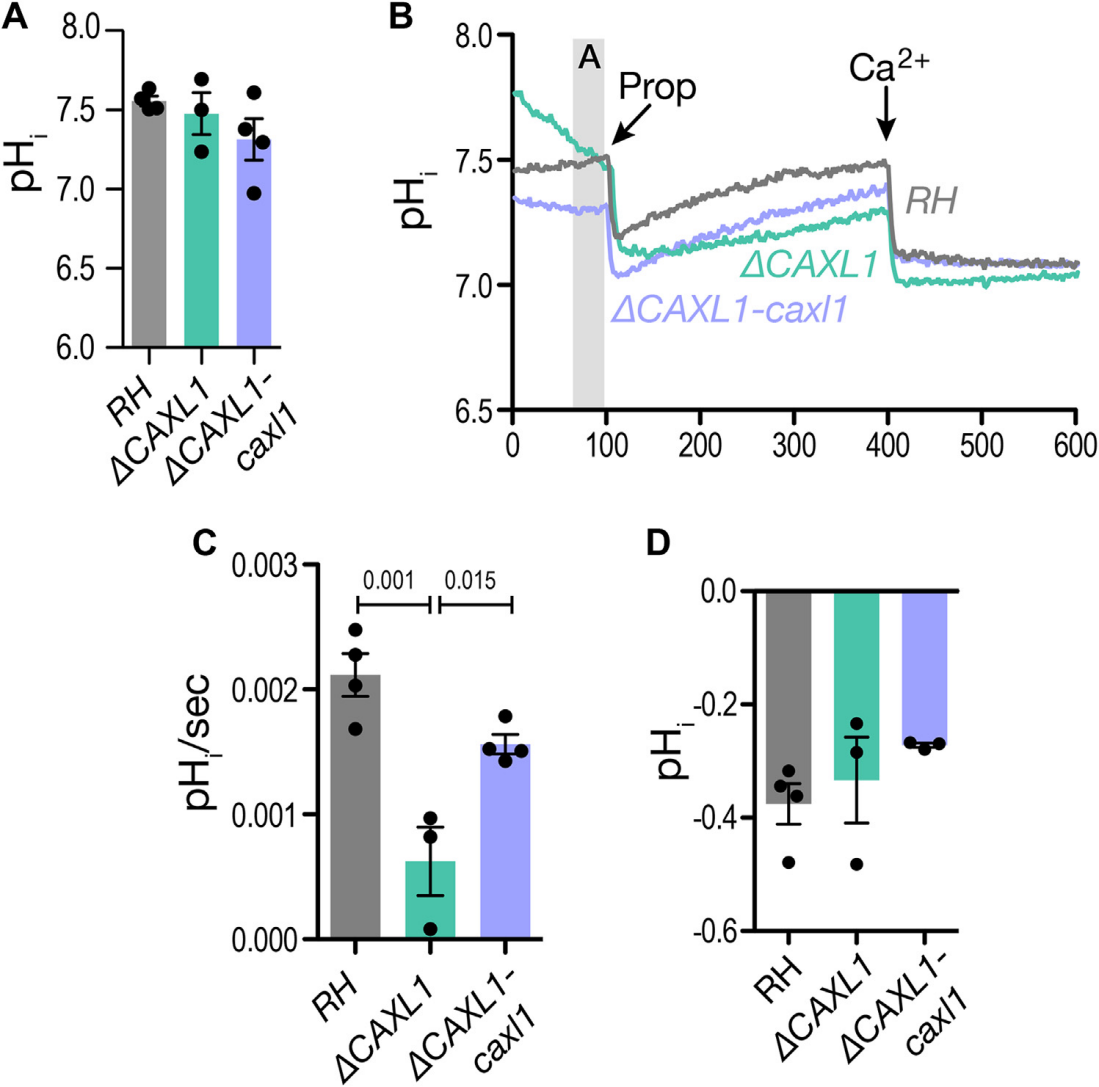
**TgCAXL1 is important for controlling cytosolic pH changes.***A*, *T. gondii* tachyzoites loaded with BCECF were suspended in a calcium-free Standard buffer with 100 μM EGTA (see Experimental procedures). pH was calculated from a standard curve described in Experimental procedures (Fig. S7*J*). The bar graph shows the average of resting pH levels. *B*, a similar experimental set-up to (*A*) but at 100 s, 3 mM sodium propionate was added, followed by the addition of 1.8 mM Ca^2+^ at 400 s. *C*, quantification of the slope after the addition of propionic acid. The slope was calculated between 100 and 120 s. *D*, change in pH after addition of Ca^2+^ at 400 s. Slopes were analyzed for RH, *ΔCAXL1*, and *ΔCAXL1-caxl1*. Values indicate mean ± SEM and are analyzed using one-way ANOVA. Prop = propionic acid.

To investigate the role of TgCAXL1 in pH regulation, we loaded parasites with the chemical pH indicator 2′,7′-bis-(2-carboxyethyl)-5-(and-6)-carboxyfluorescein (BCECF) (54). Intracellular pH of the parasite is tightly controlled through the transport of H^+^ across different membranes (55, 56). The cytoplasmic resting pH of the *ΔCAXL1* mutant was similar to the cytoplasmic pH of the parental cell line RH (Fig. 7*A*). Next, we challenged the parasites by exposing them to acidic pH through the addition of propionic acid, a weak acid, to the suspension (Fig. 7*B*). As expected, the addition of propionic acid caused cytosolic acidification. However, healthy cells activate recovery mechanisms, like the V-H^+^-ATPase and other transporters, to restore cytoplasmic pH to normal values. Both, the RH control and the *ΔCAXL1-caxl* complemented parasites were able to recover to neutral pH (Fig. 7*B*). However, the *ΔCAXL1* mutant displayed a significantly slower recovery rate, with nearly 50% reduction observed between 110 and 200 s (Fig. 7, *B* and *C*), although they were eventually able to reach neutral pH.

We noticed that the addition of extracellular Ca^2+^ to the parasites led to a drop in pH, suggesting that cytosolic Ca^2+^ influx is accompanied by proton influx, likely from acidic stores or the ER, as the extracellular buffer is not acidic. This could be through the activation of Ca^2+^/H^+^ activites at the membrane of acidic stores or activation of the SERCA activity which is known to countertransport H^+^s to keep the ER neutral (57). There was no difference in the extent of acidification by Ca^2+^ between *ΔCAXL1* and WT, suggesting that this acidification is mostly mediated by to other Ca^2+^/H^+^ exchangers and ATPases like TgA1 (Fig. 7*D*).

We tested NH_4_Cl, a weak base, that dissipates the lysosomal pH and increases cytosolic pH, but we did not observe any differences in the response between *ΔCAXL1* and the control. Both alkalinization and subsequent recovery of cytosolic pH after ammonium chloride addition (Fig. S7, *A*–*C*) were comparable between WT and *ΔCAXL1*. We also observed that GPN alkalinized the cytosol as was previously reported to act as a weak base (58), and this effect was also similar between WT and *ΔCAXL1* (Fig. S7, *D*–*F*). This suggests that proton pumps or other exchangers acting at the membrane of acidic compartments may play a more significant role in recovering the cytosolic pH from alkalinization stress.

We next investigated if TgCAXL1 may indirectly impact the pH of acidic stores, or PLVAC, and for this, we used the acidotropic pH probe, LysoSensor, that accumulates in acidic stores as it becomes protonated. An increase in fluorescence indicates an increase in proton levels, while a decrease indicates reduced proton levels. Parasites incubated with LysoSensor were analyzed by microscopy flow cytometry (ImageStream) allowing us to estimate the pH of acidic organelles in *T. gondii* (Fig. S7, *G* and *H*). At neutral pH, no significance difference in pH levels of acidic organelles was observed (Fig. S7*I*). The addition of GPN, caused a decrease in fluorescence, indicating that the Ca^2+^ released from the store in response to GPN is compensated by a Ca^2+^/H^+^ exchange activity, resulting in a decrease of protons in the acidic organelle. However, there was no difference in the change in fluorescence between RH and *ΔCAXL1*. Bafilomycin A (Baf A), a Vacuolar H^+^-ATPase inhibitor, inhibited cytosolic pH recovery after acidic stress but it did not alter the pH levels of acidic compartments in RH (Fig. S7*I*), which could be because of other modes of acidification of the acidic compartments, such as the *T. gondii* vacuolar H^+^-pyrophosphatase TgVP1 (59, 60) or TgCAXL1. Interestingly, we observed a difference in the pH of acidic stores between *ΔCAXL1* treated and not treated with Baf A, supporting some contribution of TgCAXL1 to acidification of acidic stores (Fig. S7*I*), which only becomes evident after inhibiting the V-H^+^-ATPase with bafilomycin. Thapsigargin was tested as a control, since it targets the ER, and did not affect acidification of the acidic stores (Fig. S7*I*).

In summary, TgCAXL1 appears to act as a backup mechanism for protecting cytosolic pH (after the V-H^+^-ATPase). TgCAXL1 appears to function at acidic stores by exchanging H^+^ for Ca^2+^ and this activity becomes relevant under acidic stress. The function of TgCAXL1 impacts the operation of other activities that involve H^+^ exchange like the activity of TgSERCA which is regulated by pH.

## Discussion

In this work, we characterized a *T. gondii* Ca^2+^/H^+^ exchanger or TgCAXL1, a member of a novel family of CAX exchangers. We demonstrated that TgCAXL1 functions as a Ca^2+^/H^+^ exchanger by expression of the *T. gondii* gene in yeast mutants, which are highly sensitive to high Ca^2+^ because of deletion of the yeast ortholog ScGdt1 (TMEM165) (26). Expression of TgCAXL1 restored the growth of the yeast mutant at high Ca^2+^ validating the Ca^2+^/H^+^ exchange function of TgCAXL1.

TgCAXL1 localized to the Golgi apparatus and the ER of *T. gondii* and deletion of the gene resulted in reduced release of Ca^2+^ from the ER in response to thapsigargin and diminished Ca^2+^ release from acidic stores in response to GPN. The most striking phenotype of the *ΔCAXL* mutant was the reduced ER SERCA Ca^2+^ pumping activity. The *ΔCAXL1* mutant showed normal cytoplasmic pH but were slow in recovering normal pH from an imposed acid stress. The Ca^2+^ stores where TgCAXL localizes are important for the stimulation of invasion as the *ΔCAXL1* was significantly defective in invasion. However, growth and replication were not affected in the mutant indicating that either the role of TgCAXL in intracellular parasites was minimal or it was compensated by other calcium-related functions.

The UPF0016 CAX protein family differs from previously characterized calcium/proton exchangers. Canonical CAX proteins of the Ca^2+^/cation superfamily contain 10 to 12 transmembrane domains, tend to localize to vacuoles, and are divergent from the UPF0016 subfamily (20). An in-depth phylogenetic analysis of UPF0016 members from various groups of life including metazoans, fungi, plants, bacteria and one protist, *Paramecium tetraurelia* (*P. tetraurelia*) (25), grouped into 4 different subfamilies. The alveolate protist *P. tetraurelia* encodes 4 UPF0016 paralogs that cluster together and create their own subfamily. The phylogenetic analysis supported that TgCAXL1 is part of the UPF0016 new family of CAX proteins, a finding reinforced by its localization to the Golgi apparatus, similar to other known UPF0016 proteins. The human ortholog of UPF0016 (24), as well as those in yeast (26) and the kinetoplastid *Trypanosoma cruzi* (61), also localize to the Golgi.

It has been demonstrated that the Golgi possesses the capacity to store Ca^2+^ (62) and plays a role in the sorting, processing, and glycosylation of target proteins (63). The Golgi Ca^2+^ signaling toolkit includes various ATPases and Ca^2+^ binding proteins, with expression of these proteins being non-homogeneous across its compartments. The Golgi consists of folded membrane stacks, divided into cis, medial and trans Golgi and is polarized in composition and function (64). In mammalian cells, the Golgi shares with the ER, the expression of specific proteins including SERCA (65), IP_3_, and Ryanodine receptors (62, 66), and several Ca^2+^-binding proteins. However, it is unique in the expression of the secretory protein Ca^2+^ ATPase (SPCA) (67, 68), TMEM165, and the Ca^2+^ binding proteins Calnuc (69) and Cab45 (70). Unlike the mammalian Golgi, the *T. gondii* Golgi is composed of a single stack (36). The *T. gondii* Golgi expresses various proteins involved in protein trafficking, maturation, and sorting to downstream organelles (71, 72, 73, 74, 75). The distribution of TgCAXL1 and TgSERCA suggests overlap between the functions of the Golgi and ER.

The complementation of the yeast ortholog, ScGdt1, with TgCAXL1 further supported the function of TgCAXL1 as a member of the UPF0016 subfamily. The only other calcium/proton exchanger described in yeast is the vacuolar calcium/proton exchanger Vcx1. However, deletion of Vcx1 does not cause sensitivity to high Ca^2+^ concentrations (26), indicating that this phenotype is specific to the absence of ScGDT1. ScGDT1 modulates Ca^2+^ and manganese homeostasis at the Golgi. These ions are crucial for Golgi-dependent processing and glycosylation of proteins. The ScGDT1 mutant displays a glycosylation defect of glucanosyltransferase, a marker of N and O-linked glycosylation when grown at high Ca^2+^ concentrations (28). Interestingly, complementation of the yeast GDT1 mutant with TgCAXL1 resulted in increased growth compared to the complementation with the yeast gene GDT1. This could be due either to a higher level of expression or the specific activity of TgCAXL1, which could be more efficient at transporting Ca^2+^ than Mn^2+^, making it more effective at rescuing the Calcium sensitivity of the yeast mutant (41).

According to our data, TgCAXL1 plays a critical role in mediating ER Ca^2+^ signaling through its interaction with TgSERCA. In extracellular tachyzoites, we observed reduced Ca^2+^ leakage into the cytosol induced by thapsigargin inhibition of TgSERCA. This suggests that TgCAXL1 may impact the amount of Ca^2+^ stored in the ER and Golgi, or it may be directly involved with one of the mechanisms of ER Ca^2+^ release/leakage. We confirmed the interaction of TgCAXL1 with SERCA Ca^2+^ uptake with MagFluo4 (18, 50) loaded parasites. Addition of MgATP to permeabilized parasites activated Ca^2+^ influx into TgSERCA expressing stores. TgSERCA-driven ER Ca^2+^ uptake was significantly reduced in the *ΔCAXL1* mutant, indicating that TgSERCA requires TgCAXL1 to function properly. The interdependence of Ca^2+^-ATPase and UPF0016 calcium/proton exchanger was previously demonstrated in yeast with the Type II Golgi-localized Ca^2+^-ATPase, or ScPmr1 (76), which co-localizes with the yeast orthologue, ScGDT1 (26, 27). Aqueroin was used to measure cytosolic levels of Ca^2+^ in yeast, and the *Pmr1Δ* mutant had higher resting levels of Ca^2+^ due to the inability of the cell to properly store Ca^2+^ in the Golgi. In the double *Pmr1Δ/GDT1Δ* mutant, an even higher resting cytosolic Ca^2+^ level was observed indicating that GDT1 contributes to Ca^2+^ influx into the Golgi. This interaction was further supported in yeast growth assays at high Ca^2+^ concentrations (26). While both single mutants of ScGdt1 and ScPmr1 displayed a growth defect at high Ca^2+^ concentrations, the double mutant grew at an even slower rate. Whether TgCAXL1 directly or indirectly controls TgSERCA function remains to be demonstrated. TgCAXL1 could be modulating the activity of other ER-specific proteins important for ER Ca^2+^ pumping; however, TgSERCA is the only one so far identified. In addition, no additional interactors of UPF0016 members have been biochemically identified. The resting cytosolic concentration of Ca^2+^ of the *ΔCAXL1* mutant was not different from that of the parental cells, suggesting that other mechanisms, such as the plasma membrane-localized Ca^2+^-ATPase (TgA1) (19) or uptake by other stores, may be compensating for the regulation of cytosolic Ca^2+^ homeostasis (12).

We report for the first time that pH can impact TgSERCA activity in *T. gondii*. SERCA is part of the Type II family of Ca^2+^ ATPases, which primarily transport Ca^2+^ (76). SERCA is responsible for pumping Ca^2+^ into the ER lumen, however, it has also been reported that for the ER to maintain its neutral electrochemical membrane, potassium, magnesium or hydrogen need to be countertransported across the ER membrane (77). The SERCA protein undergoes a cycle, transitioning from the E1 phase, where SERCA is facing the cytosol and its affinity for Ca^2+^ is highest, to the E2 phase, where it faces the ER lumen and its affinity for Ca^2+^ is low (77, 78). At this low Ca^2+^ affinity state, SERCA will bind to two hydrogen ions and will translocate them from the ER lumen to the cytosol. Because of this cycle, the SERCA has been described as possessing calcium proton exchange activity (52). Alkaline pH was first shown to impact SERCA Ca^2+^ uptake by Yu *et al.* in reconstituted proteoliposomes (79). Ca^2+^/proton countertransport activity was also assessed by measuring the amount of protons ejected into the cytosol by SERCA when the proteoliposome lumens were maintained at pH 6, 7, and 8. Proton ejection was highest at pH 6 and lowest at pH 8. The Ca^2+^ binding sites on SERCA are partly comprised of acidic residues, glutamic and aspartic acid (80). It was hypothesized, that when facing the lumen, a specific concentration of protons is required to facilitate Ca^2+^ dissociation and proton replacement at the Ca^2+^ binding site (52, 81). An alkaline pH could potentially inhibit this process and ultimately prevent the transition from the E2 to the E1 phase. Our results showed that TgSERCA activity is drastically affected by the pH of the medium. It is likely that amino acid residues directly or indirectly involved in Ca^2+^ binding must be sufficiently deprotonated to bind Ca^2+^ and/or transport Ca^2+^ to the ER lumen of *T. gondii*. Additionally, an alkaline pH in the ER lumen may prevent the SERCA pump from transitioning from the E2 phase (lumen side) to the E1 phase (cytoplasm side), since it requires proton binding to ensure this transition. Ultimately, protons are necessary to bind to the SERCA pump when it faces the ER lumen, enabling the transition to the cytosol to bind calcium. An acidic pH could also inhibit the pump's ability to efficiently bind calcium explaining the reduced activity that we observed at pH 6.5. A large calcium gradient exists between the cytosol and the lumen of the stores and the calcium/proton exchange activity of TgCAXL1 will be dictated by this calcium gradient, since the pH between the lumen of the stores and the cytosol is most likely neutral. In this scenario, TgCAXL1 would exchange calcium ions for protons in the membrane of the Golgi and the endoplasmic reticulum, supplying protons to SERCA in the ER lumen as it transitions from the E2 to E1 phase (Fig. S8). However, TgCAXL1 is not an essential gene suggesting that another unknown player with cation/proton exchange activity may be expressed on the ER and/or Golgi membrane. The defect in ER/Golgi Ca^2+^ uptake of the *ΔCAXL1* at pH 6.5 and 7 could be because the proper amount of protons is not being distributed around the TgSERCA protein.

TgCAXL1 mediates pH regulation in *T. gondii*. The exact function of UPF0016 proteins in proton exchange and pH modulation is not very clear. The lysosomes of individuals with mutations in TMEM165 appeared to be more acidic (26) and the same result was detected in *in vitro* experiments with TMEM165-deficient HeLa cells (26). Experiments with GDT1 heterologously expressed at the plasma membrane of *L. lactis* showed that alkaline pH facilitated Ca^2+^ influx due to the establishment of a proton gradient. This supported ScGDT1's activity as a Ca^2+^/proton exchanger (27). Modulation of pH was also tested in *A. thaliana* CCHA1 mutants, with CCHA1 being a UPF0016 homolog (82). *A. thaliana* CCHA1 mutant growth was significantly defective at both alkaline and acidic pHs compared to WT. These studies also showed that CCHA1 affects the pH of stomata cells, a type of cell that facilitates gas and liquid transport in plants (83). Acid stress experiments with the *ΔCAXL1* mutant demonstrated that TgCAXL1 guards the cytosolic pH of the *T. gondii* tachyzoite and it is part of the recovery mechanisms triggered after any cytosolic pH change. Other mechanisms in place would be the vacuolar H^+^-ATPase that pumps protons from the cytosol into acidic stores or outside the cell (56). TgCAXL1 could represent an additional mechanim of proton import into the Golgi and/or ER or it may help regulate the activity of the vacuolar H^+^-ATPase. Alkaline stress induces differentation of tachyzoites into bradyzoites in culture, but the molecular mechanism behind this phenomenon is unknown (84, 85). Future work could investigate the response of the *ΔCAXL1* mutant (generated in cytogenic strains) to pH triggers that induce differentiation or its ability to form cysts in infected mice.

The release of Ca^2+^ from GPN-targeted organelles is significantly diminished in the *ΔCAXL1* mutant. In *T. gondii*, GPN is hypothesized to release Ca^2+^ from acidic stores most likely the PLVAC (15, 86). Here we showed that after filling the stores with Ca^2+^, the Ca^2+^ efflux induced by adding GPN is significantly reduced in the *ΔCAXL1* mutants, indicating that either TgCAXL1 mediates Ca^2+^ uptake into acidic organelles or that the acidic stores are insufficiently filled with Ca^2+^ due to the lower activity of SERCA. We recently demonstrated that the activity of TgSERCA was important not only for the filling of the ER with Ca^2+^ but also for acidic stores and we proposed that this was because of the transfer of Ca^2+^ from the ER to other organelles (18). Another explanation would be that GPN is acting on the Golgi Ca^2+^ store. Recent work by Atakpa *et al.* (58) provides evidence that GPN-induced Ca^2+^ release might actually come from the ER and that the Ca^2+^ from the ER might be transported to the lysosome. Recently, another study refuted this claim and reported that GPN targets Ca^2+^ release from the lysosomes (49). Further studies are needed to determine the mechanism by which TgCAXL1 impacts the filling of acidic stores.

The comparable response to zaprinast of the *ΔCAXL1* mutant and the parental strain was puzzling, considering that Zaprinast is thought to act on neutral stores. However, our results with GPN and zaprinast addition showed that the addition of GPN impacted the Zaprinast response, indicating that the store from which zaprinast acts is affected by the lysosomotropic effect of GPN. Note that we previously reported that the zaprinast rate of Ca^2+^ release was not affected by GPN addition (45). In the present experiments, we measured the increase in the ratio and the area under the curve (the amount of Ca^2+^) and used a higher concentration of GPN (40 vs 20 μM). Previous data supported that GPN released Ca^2+^ from the PLVAC (15), but TgCAXL1 did not convincingly localize to this organelle. The diminished effect of GPN on the Δ*CAXL1* mutant could be due to the role of CAXL1 in the Golgi but also to diminished Ca^2+^ in the PLVAC as we showed that the ER is capable of transferring Ca^2+^ to other organelles through membrane contact sites (18). Therefore, the PLVAC Ca^2+^ would be impacted by the lower activity of SERCA in the ER of the Δ*CAXL1* mutant. The resting cytosolic Ca^2+^ did not change in the *ΔCAXL1* mutant which means that either the plasma membrane or other compartments could be taking up more Ca^2+^ as the SERCA is not optimally working. The lack of difference in the effect of zaprinast could result from it affecting multiple compartments, with the total stored Ca^2+^ being similar in the control and mutant. However, deletion of TgCAXL may cause an alteration in the distribution of stored Ca^2+^. Further experiments would be needed expressing genetic pH and Ca^2+^ indicators in the largest acidic store in *T. gondii*, the PLVAC, as well as the mitochondrion and the apicoplast, to understand the interplay and communication between these Ca^2+^ stores.

TgCAXL1 is a calcium/proton exchanger dually localized to the ER and Golgi apparatus of *T. gondii* and for the first time, we showed that the Golgi apparatus is involved in mediating Ca^2+^ signaling in *T. gondii*. Regulating pH levels in the cytosol and in intracellular stores is critical for protein activity, specifically TgSERCA at the ER membrane. Disrupting the tight regulation of protons and Ca^2+^ impacted the parasite's ability to invade host cells, an essential step of the lytic cycle. The modest growth phenotype of the *ΔCAXL1* mutant could be explained by the expression of redundant molecules with similar functions such as the TgCAXL1 paralog, TgGT1_251400. TgGT1_251400 contains the two canonical motifs unique to members of the UPF0016 calcium/proton exchanger family and consists of six transmembrane domains (Protter). Further studies on TgGT1_251400 are necessary to determine whether it plays a role in Golgi calcium signaling. Furthermore, if TgGT1_251400 possesses similar function to TgCAXL1, generating a double knockout mutant of TgGT1_251400 and TgCAXL1 might drastically affect the parasite's ability to undergo the lytic cycle.

This work contributes to our understanding of the Ca^2+^ signaling toolkit of *T. gondii* and how some of these players may influence specific steps of the parasite lytic cycle.

## Experimental procedures

### Cell cultures

*T. gondii* RH type I and *TATiΔKu80* (35) tachyzoites were utilized. Parasites were grown in human telomerase reverse transcriptase (hTERT) confluent monolayers in Dulbecco's Modified Eagle Medium with high glucose (DMEM-HG) supplemented with 1% bovine calf serum (BCS) (GemCell GeminiBio) as previously described (60). For replication and invasion experiments, parasites were grown in human foreskin fibroblasts (HFF) in DMEM-HG + 1% BCS.

### Phylogenetic analysis

The phylogenetic tree constructed by Demaeged *et al.* (25) was often referenced for annotated UPF0016 proteins from eukaryotes to prokaryotes. Updated protein sequences and names were obtained from the National Center for Biotechnology Information protein (NCBI) database. Apicomplexan sequences were identified in the ToxoDB database by Orthomcl.org. Kinetoplastid sequences were retrieved using the Basic Local Alignment Search tool for proteins (BlastP) from the TriTryp database. Sequences were aligned using phylogeney.fr platform (87), with the ClustalW program selected for the alignment (88, 89). A maximum likelihood tree was constructed using MEGA, with the LG substitution model and a discrete gamma distribution (G), with a bootstrap of 1000 (90). Table S1 lists the accession number of the sequences used in the analysis.

### Epitope tagging and generation of mutants

Development of parasite mutants was achieved using CRISPR/Cas9 mediated gene disruption (32, 33). For C-terminal epitope tagging, the CRISPR/Cas9 plasmid consisted of a 20 bp sgRNA homologous to the C-terminus of the *TgCAXL1* gene (TgGT1 319,550) locus (amplified with AC107 and AC119, all primers are listed in Table S2). The repair template, consisting of three copies of a human influenza hemagglutinin (3xHA tag) and the chloramphenicol (CAT) drug cassette was amplified from the 3xHA-pLIC-CAT (91) plasmid with primers (AC 105 and 106) that contain 35 to 40 bp of homologous DNA to TgCAXL1. *TATiΔku80* parasites were transfected with the tagging construct alongside the CRISPR/Cas9 plasmid. Parasites were selected with 20 μM chloramphenicol and serially diluted for single-cell subcloning. Clones were validated by PCR, immunofluorescence assay (IFA), and western blots. Anti-HA antibody (1:1000) was used to detect CAXL1-smHA expression and anti-tubulin (1:10,000) as loading control. To generate TgCAXL1-smHA parasites, we followed the same approach used to generate the TgCAXL1-3xHA, with the modification of tagging the C-terminus of the *TgCAXL1* gene with 10 copies of a human influenza hemagglutinin (HA) tag (smHA) and a chloramphenicol (CAT) drug cassette. The smHA and CAT cassette were amplified from the smHA-pLIC-CAT plasmid (37, 91) with primers, AC105 and AC106. TgCAXL1-smHA was validated using the same methods as TgCAXL1-3xHA.

To generate the *ΔCAXL1* mutant, 1 kb of homologous DNA flanking the 5′ and 3′ UTR of the *TgCAXL1* gene was inserted upstream and downstream of the dihydrofolate reductase (DHFR) cassette, respectively. The transfection insert was amplified from the newly constructed plasmid using primers AC178 and AC179. To enhance the likelihood of obtaining a clean deletion mutant, two CRISPR/Cas9 plasmids were generated, one with the sgRNA specific for the 5′ end (using primers AC163 and AC119) and another for the 3′ end (using primers AC164 and AC119). The repair template and both plasmids were transfected in RH type I parasites and selected with 1 μM pyrimethamine for two passages. The mixed population was serially diluted to select clonal populations for further analysis. Clones were validated *via* PCR and quantitative real-time PCR. Complementation of *ΔCAXL1* was achieved by transfecting *ΔCAXL1* with a plasmid containing the TgCAXL1 cDNA, fused to a 3xHA at the c-terminal end and expressed under the TgCAXL1 native promoter, which included 1000 bp of the TgCAXL1 5′ UTR. Parasites were selected with 20 μM chloramphenicol and serially diluted to isolate a clonal population. Clonal lines of the complemented mutant *(ΔCAXL1-caxl1*) were verified by PCR (using primers AC 292+293 and AC76 and AC215), western blots, and quantitative real time-PCR. All mutants were transfected with a plasmid containing the sequence for the tdTomato gene (a gift from Boris Striepen) (39) and were further selected by FACS sorting. Single red parasite clones were used for the experiments.

### Immunofluorescence assay

Parasites were isolated from 70% host celllysed cultures and were filtered through a 5 μm nuclepore membrane and washed once with buffer A with glucose (BAG) (116 mM NaCl, 5.4 mM KCl, 0.8 mM MgSO_4_•7H_2_O, 50 mM HEPES, 5.5 mM Glucose, pH adjusted to 7.3). Parasites were adhered to glass slides using poly-L-lysine, fixed with 3% paraformaldehyde, permeabilized with 0.3% triton-X-100, and blocked with 3% bovine serum albumin (BSA) made in 1× PBS pH 8. Parasites were incubated with the primary antibody for 1 h at room temperature, washed with PBS (pH 8), and then incubated with the secondary antibody for 1 h in the dark. The slides were washed 4× with PBS (pH 8). Coverslips with parasites were mounted onto slides using Fluoromount-G and 1% DAPI.

Intracellular parasites were stained in the same way; however, host cells were prepared on sterile coverslips and allowed to grow for 24 h before being infected. Parasites were then allowed to grow for an additional 24 h. To probe for the Golgi-apparatus (36), parasites were transfected with 50 μg of GRASP55-RFP plasmid and seeded onto a coverslip containing host cells made in advance. Rabbit anti-RFP was used at a 1:1000 dilution to detect GRASP55. To probe for the ER, we used anti-SERCA at 1:500. This antibody was generated in the lab against the recombinant SERCA and was validated by western blots and IFAs (18). Mouse anti-HA was used at 1:1000 and Rat anti-HA was used at 1:25. Rabbit anti-VP1 was used at 1:1500 (15) and mouse anti-ZnTpr (Zinc transporter) was used at 1:200 (92). Secondary antibodies consisted of Invitrogen Alexa Fluor 488 Goat anti-Mouse and Goat anti-Rat, 546 Goat Anti-Rat and Goat Anti-Rabbit all at 1:1000 dilutions. Slides were visualized with the Olympus IX71 inverted microscope using the DeltaVision imaging system. Images were deconvolved and analyzed with softWoRx software as previously (93).

### qRT-PCR

RNA was extracted from parasites using TRIzol Reagent (Ambion-Life Technologies) as directed by the product manual and treated with 1 μl of DNase I (NEB) for 10 min at 37 °C. cDNA synthesis was performed using the Superscript III First Strand System from Invitrogen and diluted to a working stock of 100 ng/μl. Transcript levels were detected by iQ Syber Green supermix with primers AC214 and AC215 (Table S2) targeting the first exon of TgCAXL1. 100 ng of cDNA was used for each assay reaction. Samples were run in a CFX96 Real Time PCR System (Bio-Rad). All values were analyzed using the BioRad CFX manager program to determine the relative expression level of TgCAXL1 in all cell lines. TgCAXL1 expression level in RH was set to 1 and actin (TgGT1_209030) and tubulin (TgGT1_316400B) were used as housekeeping controls (Tubulin amplified with primers AC327 and AC328 and actin amplified with primers AC329 and AC330) (Table S2).

### Yeast growth assays

*GDT1Δ*+EV, *GDT1Δ*+GDT1, and *GDT1Δ* yeast strains and the pRS416 plasmid were a gift from Dr Roberto Docampo, who obtained them from Dr Pierre Morsomme at the Université Catholique de Louvain in Belgium (26). The pRS416 plasmid (26), consisting of the TP1 (triose phosphate isomerase 1) promoter and a URA3 metabolic marker, was used as the complementation vector. *TgCAXL1* cDNA was fused to 69 bp of the GDT1 signal peptide by Twist Bioscience and cloned into the pRS416 plasmid downstream to the TP1 promoter using Restriction Enzymes XhoI and EcoRI (NEB). The pRS416-CAXL1 plasmid was then transformed into *GDT1Δ* and selected on a synthetic defined (SD) medium supplemented with all amino acids except uracil (0.7% Yeast Nitrogen Base without amino acids, 2% glucose and 0.77% complete supplement mixture without uracil-pH 6). For Ca^2+^ growth experiments, the media consisted of 0.2% Yeast Nitrogen Base without amino acids and ammonium sulfate, 76 mM NH_4_Cl, 2% glucose, and 0.77% complete supplement mixture (CSM) without uracil, pH 6 and 550 mM of Ca^2+^. Ca^2+^ was added after autoclaving the media to avoid precipitation. Solid media was made by adding 2% agar (BD BactoAgar, Fisher Scientific). For plate growth assays, yeast was grown overnight in SD medium without uracil at 30°C. Yeast was diluted to an OD_600_ of 1 and three 1:10 serial dilutions were made. 10 μl of each dilution were plated on solid media with or without 550 mM Ca^2+^ and allowed to grow for 4 to 6 days at 30 °C. For the liquid growth assay, yeast was grown overnight at 30 °C. After 24 h of incubation, yeast was diluted to an OD of 0.1 in SD media, with or without 550 mM of Ca^2+^. Then, 200 μl of the culture was added to each well of a 96-well plate and the OD_600_ was measured every 30 min for 48 h.

### Plaques and plaquing efficiency assays

Parasites were filtered through a 5 μm Nuclepore membrane to remove host cell debris. 200 parasites of a mutant were seeded to a confluent hTERT host cell monolayer in a 6-well plate and allowed to grow for 7 days at 37 °C (60). The monolayer was then fixed with 70% ethanol and stained with crystal violet. Plaque sizes were analyzed with ImageJ (94). Plaquing efficiency was assayed using 1000 parasites, which were allowed to infect a confluent host cell monolayer for 30 min at 37 °C. Afterwards, the cells were washed twice with sterile PBS followed by the addition of fresh media. Parasites were allowed to grow for 4 days and then fixed with 70% ethanol and stained with crystal violet. The number of plaques were counted using ImageJ.

### Replication assay

Replication assays were performed with parasites expressing RFP td-tomato. HFF cells were seeded on coverslips 24 h in advance in DMEM-HG + 15% fetal bovine serum. After 24 h, HFF cells were infected with 5 × 10^5^ of RFP-expressing parasites and allowed to grow for another 24 h in DMEM-HG + 1% bovine calf serum. After 24 h, the media was aspirated, and the coverslip was placed on a glass slide with 10 μl of 1X phosphate-buffered saline (PBS). 100 parasitophorous vacuoles (PVs), performed in duplicate per cell line, were quantified for each cell line (95).

### *T. gondii* growth assay

Growth assays were done with parasites expressing RFP td-tomato. Parasites that were 70% egressed were filtered through a 5 μm Nuclepore membrane, and serially diluted in clear DMEM-HG media supplemented in 1% BCS. 4000 parasites were seeded in a confluent 96-well plate and fluorescence was measured every day for 6 days using a SpectraMax M2 plate reader (Molecular Devices) with 544/590 nm emission/excitation wavelengths. To calculate the parasite number, a standard curve was generated by plotting the fluorescence signal produced by varying known parasite concentrations (95).

### Egress assay

Egress assays were done in 35 mm MatTek dishes. hTERT cells were seeded and allowed to grow for 24 h to reach about 75 to 90% confluency. 24 h later, 5 × 10^5^ parasites were added to the dishes and allowed to grow for another 20 to 24 h. The media was then changed to Ringer's buffer, also used as an extracellular buffer (155 mM NaCl, 3 mM KCl, 1 mM MgCl_2_, 3 mM NaH_2_PO_4_, 10 mM HEPES, pH 7.3, 5 mM glucose) (10) + 1.8 mM of Ca^2+^. Dishes were placed on the microscope in a 37 °C chamber. After 1 min of recording, 0.01% saponin was added and the video was recorded until all parasites in the field of view egressed.

### Invasion assay

Invasion was modified from the previously described protocol by Kafsack *et al.* (40, 60). Briefly, HFF cells were prepared on coverslips 24 h in advance. The following day, freshly lysed parasites were collected, spun down at 1800 RPM, and resuspended in 2 ml of freshly made invasion media (DMEM-HG supplemented with 3% FBS, 10 mM HEPES, pH at 7.4). 2 × 10^7^ parasites were resuspended in a final volume of 2 ml and added to HFF cells and placed on ice for 20 min. The plate was moved to 37 °C for 5 min and immediately placed back on ice. Slides were fixed with 3% paraformaldehyde and blocked in 10% FBS. Subsequently, slides were first incubated with the Rabbit anti-SAG1 at 1:1000, exposed to 1% Triton X-100, and then incubated with Mouse anti-SAG 1:200. Secondary antibodies used were Alexa Fluor 488 Goat anti-Mouse and Alexa Fluor 546 Goat-anti Rabbit. Slides were also stained with DAPI. 100 parasites were analyzed for each slide (two slides per cell line).

### pH and calcium measurements

All experiments were carried out in a Hitachi F-7100 fluorescence spectrophotometer. The parasite suspension was continuously stirred, and reagents were added with a Hamilton syringe at the indicated times. Parasites were collected from 70 to 80% lysed culture flasks. The host cell monolayer was gently scraped off and parasites were resuspended in BAG. The suspension was filtered through an 8 μm Nuclepore membrane to remove host cell debris.

FURA-2-AM loading was done using a previously published protocol (86, 96). Final parasite resuspension and all experiments were done in Ca^2+^ free Ringer's Buffer achieved by adding 100 μM EGTA to chelate extracellular Ca^2+^ unless otherwise mentioned. Fluorescence was detected by using 340/380 nm excitation and 510 nm emission wavelengths as previously described (86).

For BCECF experiments, parasite harvest, centrifugation, BCECF-AM loading, and fluorescence experiments were done following the previously published protocol to measure cytosolic pH in extracellular tachyzoites (56). Following parasite collection and centrifugation, 6 μM of BCECF-AM was added to the parasite suspension and incubated in a 30 °C shaking water bath for 20 min. Parasites were resuspended in BAG and fluorescence measurements (including calibration) were done in Ca^2+^ free Standard buffer (135 mM NaCl, 5 mM KCl, 1 mM MgSO_4_-H_2_O, 10 mM Hepes/Tris, and 5 mM glucose) with 100 μM of EGTA unless otherwise indicated. The BCECF fluorescence signal was detected with a 440/505 nm excitation and 530 nm emission wavelength. Intracellular pH was calculated by using the BCECF fluorescence signal from parasites suspended in a high potassium buffer (140 mM KCl, 5 mM glucose, 1 mM CaCl_2_, 1 mM MgSO_4_, 10 mM Hepes/Tris) from pH 5.5 to 8. The potassium ionophore, 5.2 μM nigericin was added and once the curve stabilized, the fluorescence signal was plotted against the pH of the buffer at which it was measured, and a standard curve was generated (55). Using this standard curve, the intracellular pH was calculated (Fig. S7*J*).

For MagFluo4 experiments (18), purified parasites were centrifuged and washed twice in BAG and the pellet was resuspended in 1 ml of BAG and centrifuged at 5000 rpm for 2 min. This final pellet was resuspended in HBS buffer (135 mM NaCl, 5.9 mM KCl, 11.6 mM HEPES, 1.5 mM CaCl_2_, 11.5 mM Dextrose, 1.2 mM MgCl_2_, pH 7.3) containing 20 μM MagFluo4-AM, 1 mg/ml bovine serum albumin, and 0.2 mg/ml Pluronic F127. Parasites with indicator were incubated at 20 °C for 1 h and gentle shaking, and the tube inverted every 10 to 15 min to prevent the parasites from settling. After the incubation, parasites were washed twice with Cytosol Like Media (CLM) (140 mM KCl, 20 mM NaCl, PIPES 20 mM, 1 mM EGTA, buffered with 10 M KOH to a final pH 7.0). The entire resuspension was then transferred to a cuvette in a final volume of 1.8 ml of CLM and placed in the fluorometer cuvette. MagFluo4 signal is detected at 485 nm excitation and 520 nm emission wavelength. After 100 s, 48.6 μM digitonin was added for 300 s to allow permeabilization. Permeabilized cells were centrifuged at 5000 rpm for 2 min twice and washed with CLM and then resuspended to a final concentration of 1 × 10^9^ parasites/ml. 2 × 10^7^ parasites were placed in a final volume of 1.95 ml of CLM+Ca^2+^ (CLM + 220 nM free Ca^2+^) in a cuvette. Maxchelator was used to calculate free calcium concentrations at each pH (https://somapp.ucdmc.ucdavis.edu/pharmacology/bers/maxchelator/). Based on those calculations the concentration of CaCl_2_ added for each pH was: 57 μM for pH 6.5, 370 μM for pH 7.0, 850 μM for pH 7.5, and 981 μM for pH 8.0.

### Lysosensor and flow cytometry

For LysoSensor experiments, parasites were collected from 80 to 90% lysed monolayers, filtered through an 8 μm Nuclepore membrane, washed in BAG, and centrifuged at 1900 rpm for 5 min twice. Parasites were resuspended at a final concentration of 1 × 10^8^ parasites/ml in BAG. Parasites were separated in 1 ml aliquots and mixed with 1 μM of LysoSensor Green DND-189 (Thermo-Fischer) for 1 h at 30 °C. Afterwards, parasites were washed twice with BAG and centrifuged for 2 min at 5000 RPM each time and then resuspended to a final concentration of 1 × 10^8^ parasites/ml in BAG for experiments. Experiments were carried out using flow cytometry (see below).

Measurement of LysoSensor fluorescence was carried out in the ImageStream X MK II (Luminex Corporation, Seattle, Washington). 200 μl of parasites resuspended at a concentration of 1 × 10^8^ parasites/ml were used for analysis. Parasites were excited at 405 nm using channel 7 and the bright field using channel 1. 10,000 events were sampled for each run. We used the Image Data Exploration and Analysis Software (Amnis) to gate and mask the intensity of fluorescent single parasites in focus to calculate the median fluorescence intensity for each run.

### Statistical analysis

The data was expressed as the mean ± SEM from at least three independent biological repeats. Statistical analyses were performed using GraphPad PRISM. Student's *t* test, one-way ANOVA, or two-way ANOVA tests were used when appropriate and indicated in the figure legends. Significance was considered when the *p*-value was less than 0.05.

## Data availability

All data are contained within the manuscript.

## Supporting information

This article contains supporting information.

## Conflict of interests

The authors declare that they have no conflicts of interest with the contents of this article.
