# Supplementary material for: The contribution of the Golgi and the endoplasmic reticulum to calcium and pH homeostasis in Toxoplasma gondii
Source: J Biol Chem. 2025 Mar 3;301(4):108372. doi: 10.1016/j.jbc.2025.108372 (PMC12131190; doi:10.1016/j.jbc.2025.108372)
Supplement: Supplemental Tables and Figures [file mmc1.pdf]

Supporting Information

The contribution of the Golgi and the endoplasmic reticulum to calcium and pH homeostasis in *Toxoplasma gondii*.

Abigail Calixto, Katherine Moen and Silvia NJ Moreno

Supplemental Tables and Figures

Table S1: Accession number of organisms used in phylogenetic analysis.

Abbreviation	Accession Number	Organism
Sce	AAS56893.1	*Saccharomyces cerevisiae*
Cal	EEQ45872.1	*Candida albicans*
Cga	XP_003194471.1	*Cryptococcus gattii*
Dre	NP_997848.1	*Danio rerio*
Sma	XP_002580560	*Schistosoma mansoni*
Ame	XP_623837.2	*Apis mellifera*
Hsa	NP_060945.2	*Homo sapiens*
Mmu	NP_035756.2	*Mus Musculus*
Cel	NP_497567.1	*Caenorhabditis elegans*
Tg*	XP_002369832.2	*Toxoplasma gondii*
Nca	XP_003880625.1	*Neospora caninum*
Bes	XP_029220247.1	*Besnoitia besnoiti*
Ete	XP_013233795.1	*E. tenella*
Tcr	XP_819449.1	*Trypanosoma_cruzi_CLB*
Lma	XP_001682608.1	*Leishmania major strain Friedlin*
Lbr	XP_001564159.1	*Leishmania braziliensis*
Saa1	AFZ34620.1	*Stanieria cyanosphaera*
Saa2	WP_015192292.1	*Stanieria cyanosphaera*
Tem1	ABG51526.1	*Trichodesmium erythraeum*
Tem2	WP_011611892	*Trichodesmium erythraeum*

Table S2. Primers used in this study

Primer name	Sequence
**C-terminal tagging**	
AC105	TTCGTCCTCTTCGCGATCTTTGGCGCCGTCCTAGATCTGTACCCGTACGACGTCCCGGA
diffdiff	GGCCGAGGTGGACTCGCAGATGTGCTCTGTGCCATTGAATTAAGCCCCGCCCTGCC
AC107	TGATCTTCTCTCGCCTCCGAGTTTTAGAGCTAGAAATAGCAAG
**KO**	
AC119	AACTTGACATCCCCATTTAC
AC178	GCAGCTTCTGCTCATTTTACGAAGTAGAC
AC179	AGAGAGCACAAGACATGCATCCAGAG
AC 163	GGGTTGCCCACCTGAAAGCGGTTTTAGAGCTAGAAATAGCAAG
AC 164	TTCGTCCTCTTCGCGATCTTGTTTTAGAGCTAGAAATAGCAAG
**Complement**	
AC 292	atgacgagacgtttaGCAGCTTCTGCTCATTTTACGAAGT
AC 293	GTAGTCCGGGACGTCGTACGGGTACCTAGGCAGATCTAGGACGG
AC 76	ATGCGCAACTGTTTGAACAGGG
AC 215	AGAAAAGCCTTCCACGAGCAT
**RT-PCR**	
AC 214	AGGCGTTCTGCTTCCTGC
AC 215	AGAAAAGCCTTCCACGAGCAT
AC 327 (Tubulin For)	GACGACGCCTTCAACACCTTCTTT
AC 328 (Tubulin Rev)	AGTTGTTCGCAGCATCCTCTTTCC
AC 329 (Actin For)	TCCACCATGAAGATCAAGGTCGTT
AC 330 (Actin Rev)	ACATCTGCTGGAAGGTGGAG

Supplemental Figures

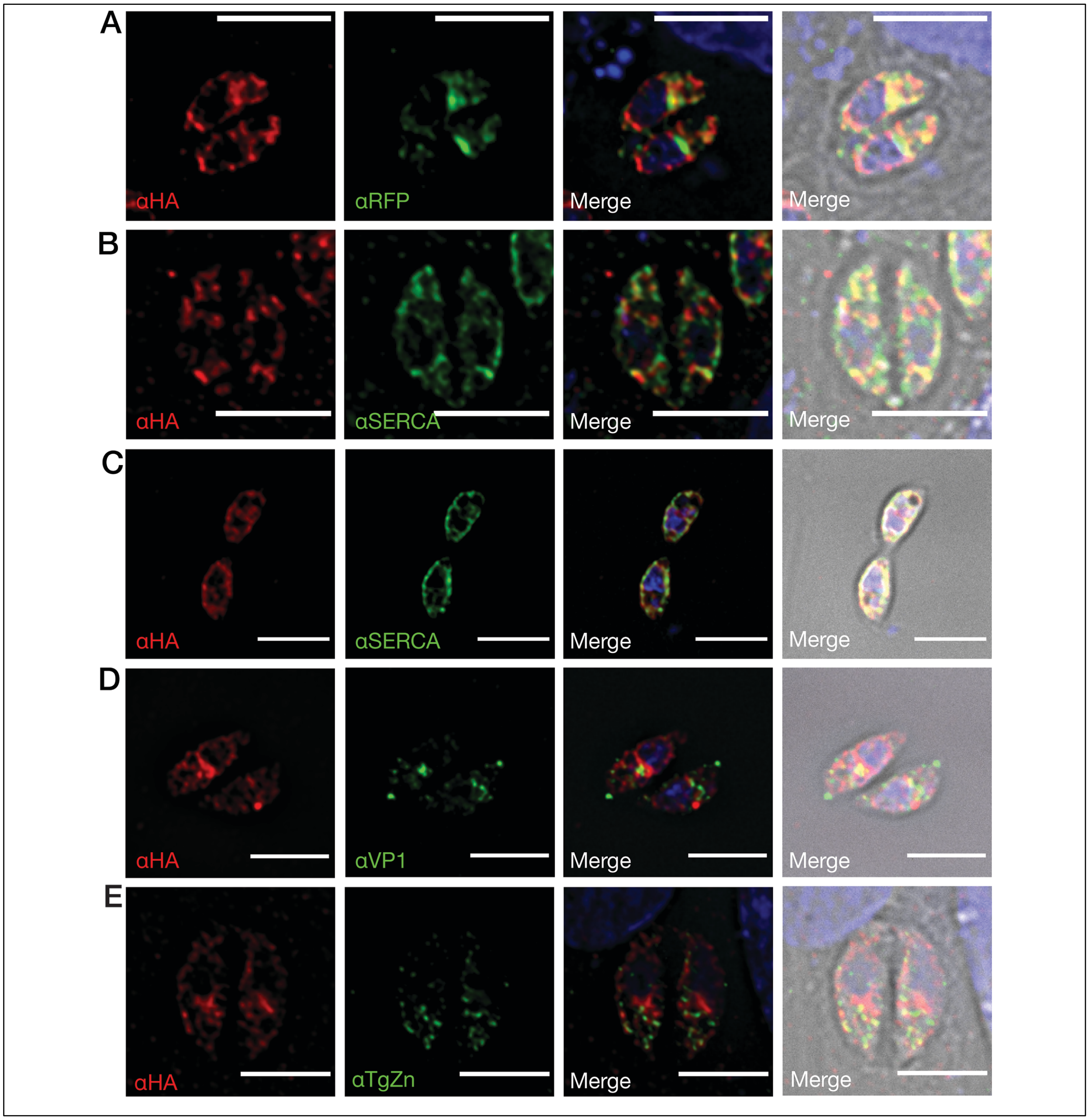

Figure S1 Localization of CAXL1-3xHA to the ER and Golgi. A) Immunofluorescence assay of intracellular CAXL1-3xHA parasites using rabbit anti-RFP (1:120) antibody labeling the Golgi marker GRASP55 (Pearson coefficient = 0.6019), B) IFAs of intracellular parasites (Pearson coefficient = 0.6299). C) IFA of extracellular tachyzoites with rat anti-HA (1:25) antibody and the ER marker, guinea pig anti-SERCA (1:500) antibody (Pearson coefficient = bottom parasite: 0.8907, and top parasite: 0.8907). D) IFAs of extracellular TgCAXL1-smHA tachyzoites with rat anti-HA (1:25) and rabbit anti-VP1 (1:1500) labeling the Plant-like vacuolar compartment (PLVAC) (Pearson coefficient = 0.4130) and E) IFAs of intracellular TgCAXL1-3HA tachyzoites with rat anti-HA (1:25) and mouse anti-ZnT (1:200) labeling the acidocalcisome and the PLVAC (Pearson coefficient = 0.4984).

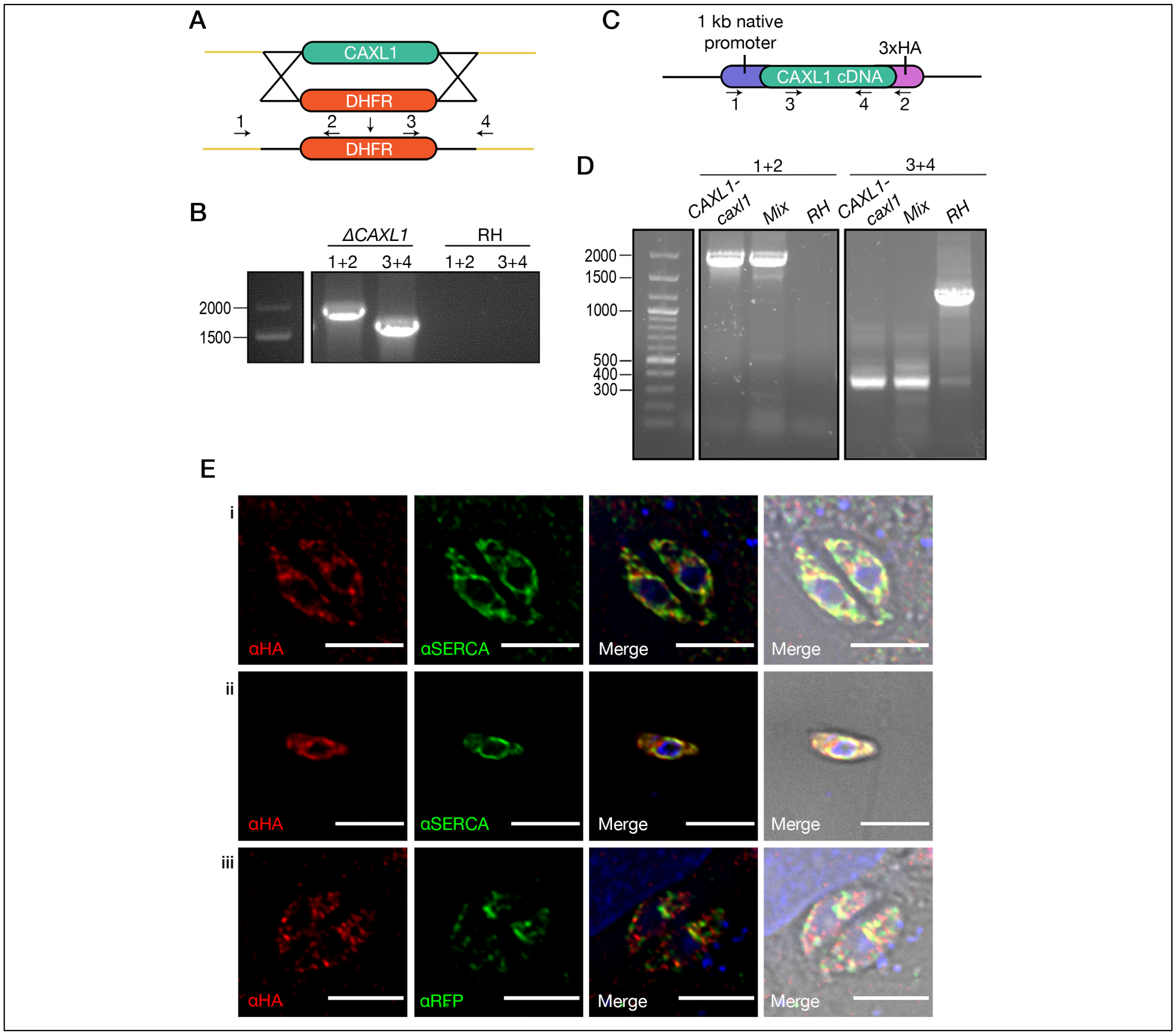

Figure S2 Validation of *ΔCAXL1* and *ΔCAXL1-caxl1*. A) Schematic of the generation of knockout of TgCAXL1 in RH Type I parasites. Numbers indicate primers used (Shown in Table S2) for the PCR validation in B. B) Genomic validation of *ΔCAXL1* using primer pairs 1+2 (AC181+AJ141; Table S2) and 3+4 (AC180+AC182; Table S2) as shown in A. Expected size of primer pair 1+2 in *ΔCAXL1* is 1.9 kb and of primer pair 3+4 in *ΔCAXL1* is 1.6 kb. C) Schematic of plasmid used to generate *ΔCAXL1-caxl1*. Numbers indicate primers used in PCR validation in D. D) Genomic validation of *ΔCAXL1-caxl1* clonal cell line using primer pairs 1+2 (AC292+AC293; Table S2) and 3+4 (AC76+AC215; Table S2) as shown in C. Mix is mixed population used as control. Expected size of primer pair 1+2 in *ΔCAXL1-caxl1* is 2 kb. Expected size of primer pair 3+4 in *ΔCAXL1-caxl1* is 441 bp and in RH wildtype parasites is 1.3 kb. E) Immunofluorescence of i) intracellular and ii) extracellular *ΔCAXL1-caxl1* parasites using rat anti-HA (1:25) antibody and the ER marker, guinea pig anti-SERCA (1:500) antibody and iii) intracellular parasites using rat anti-HA (1:25) and rabbit anti-RFP (1:120) labeling the Golgi marker GRASP.

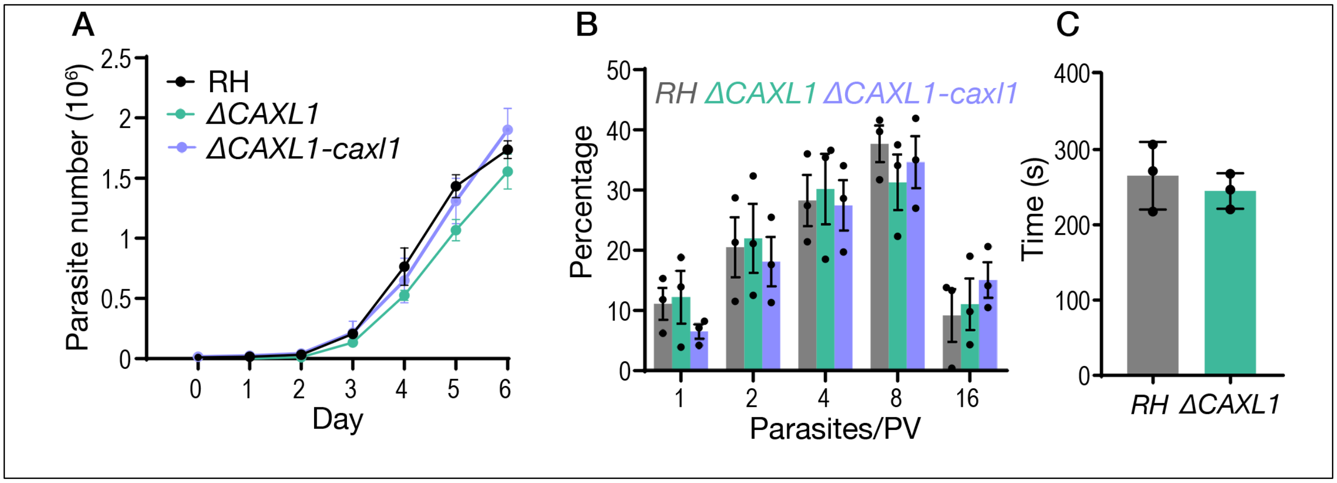

Figure S3. **Growth phenotype of *ΔCAXL1*.** A) RH, *ΔCAXL1*, and *ΔCAXL1-caxl1* expressing td-tomato were used for growth assays. Fluorescence was measured every day for 6 days and normalized to parasite number. B) Replication of RH, *ΔCAXL1*, and *ΔCAXL1-caxl1* td-tomato expressing clones. Confluent HFFs grown in a 24 well plate were infected with 5 x 10^5 parasites. At least 100 PVs were quantified. C) Egress assay. hTERT cells were grown in MatTek dishes for 24 hrs. 5 x 10^5 parasites were added to hTERT cells and allowed to grow for an additional 24 hrs. Time in seconds (s) for parasites to egress from host cells after addition of 0.01% saponin at 1 min in Ringer's buffer media containing 1.8 mM of Ca^{2+}. A, B, and C are mean values ± SEM from three independent biological replicates and each was done in duplicates. A and B were all analyzed using one-way ANOVA. C was analyzed using Student t-test.

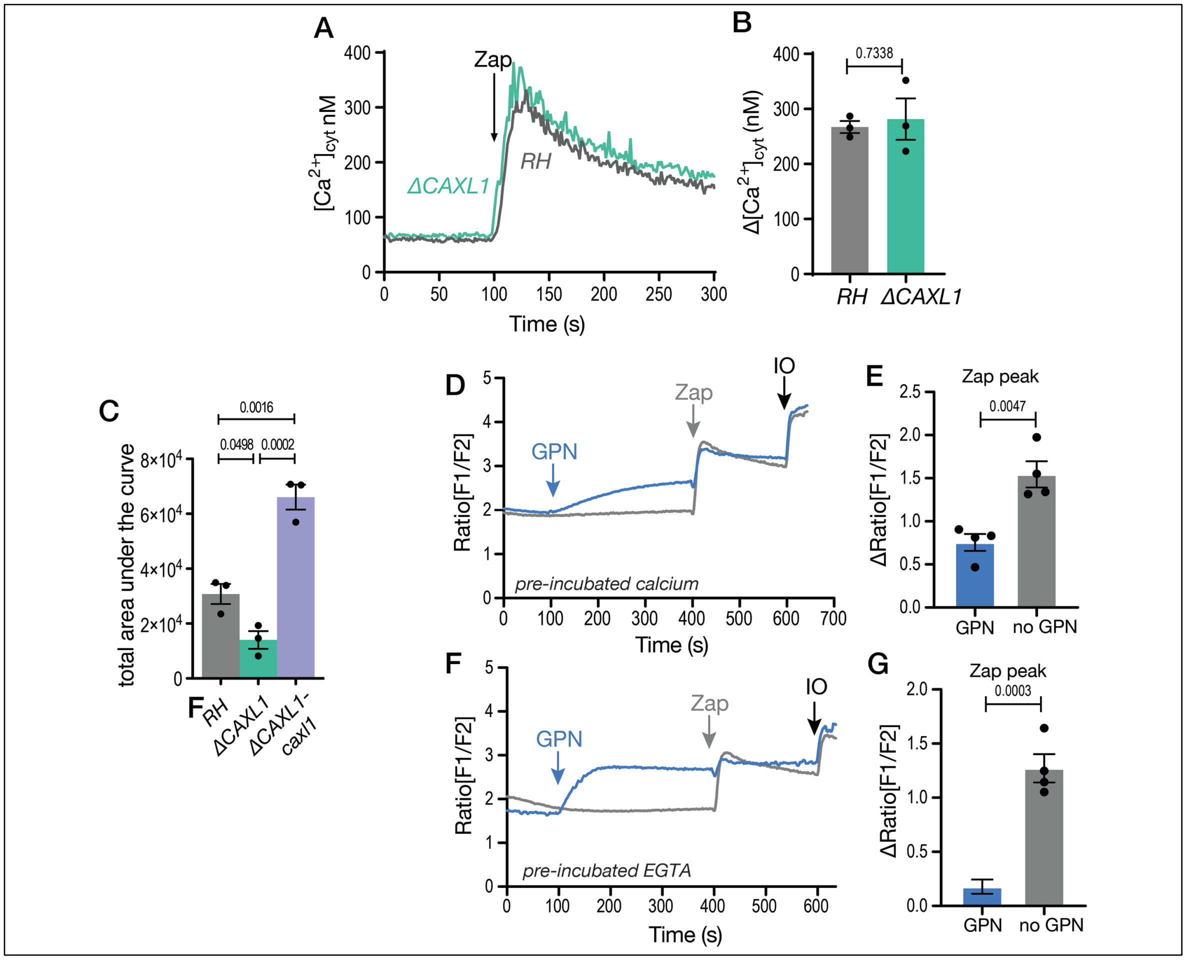

Figure S4: A) Cytosolic Ca^{2+} changes after the addition of 100 µM zaprinast at 100 sec in RH and *ΔCAXL1* parasites suspended in Ringer buffer with 100 µM EGTA. B) Bar graphs represent peak Ca^{2+} after addition of zaprinast. C) Total area under the curve for RH, *ΔCAXL1 and ΔCAXL1-caxl1* loaded with FURA 2-AM after addition of 40 µM GPN. Cells were resuspended in Ringer's buffer + 100 µM EGTA. Area under the curve was measured for 300 sec post injection of 40 µM GPN. Analysis is from Fig. 5G. D) Tachyzoites were preincubated with 1.8 mM Ca^{2+}. The gray trace shows the addition of 100 µM zaprinast at 400 sec and 1 µM ionomycin (IO) at 600 sec. The blue line shows the addition of 40 µM GPN at 100 sec, followed by 100 µM zaprinast at 400 sec and 1 µM IO at 600 sec. E) Bar graph shows the quantification of peak calcium after the addition of zaprinast by itself (no GPN, *gray bar*) compared to the peak calcium following the addition of GPN (*blue bar*) from D). F) Tachyzoites preincubated with 100 µM EGTA. The gray trace shows the addition of 100 µM zaprinast at 400 sec and 1 µM ionomycin (IO) at 600 sec. The blue trace shows the addition of 40 µM GPN at 100 sec, followed by 100 µM zaprinast at 400 sec and 1 µM IO at 600 sec. G) Bar graph shows the quantification of peak calcium after addition of zaprinast by itself (no GPN, *gray bar*) compared to the peak calcium following the addition of GPN (*blue bar*) from F). Statistical analysis was done from three independent biological experiments. E and G were analyzed using Student t-test.

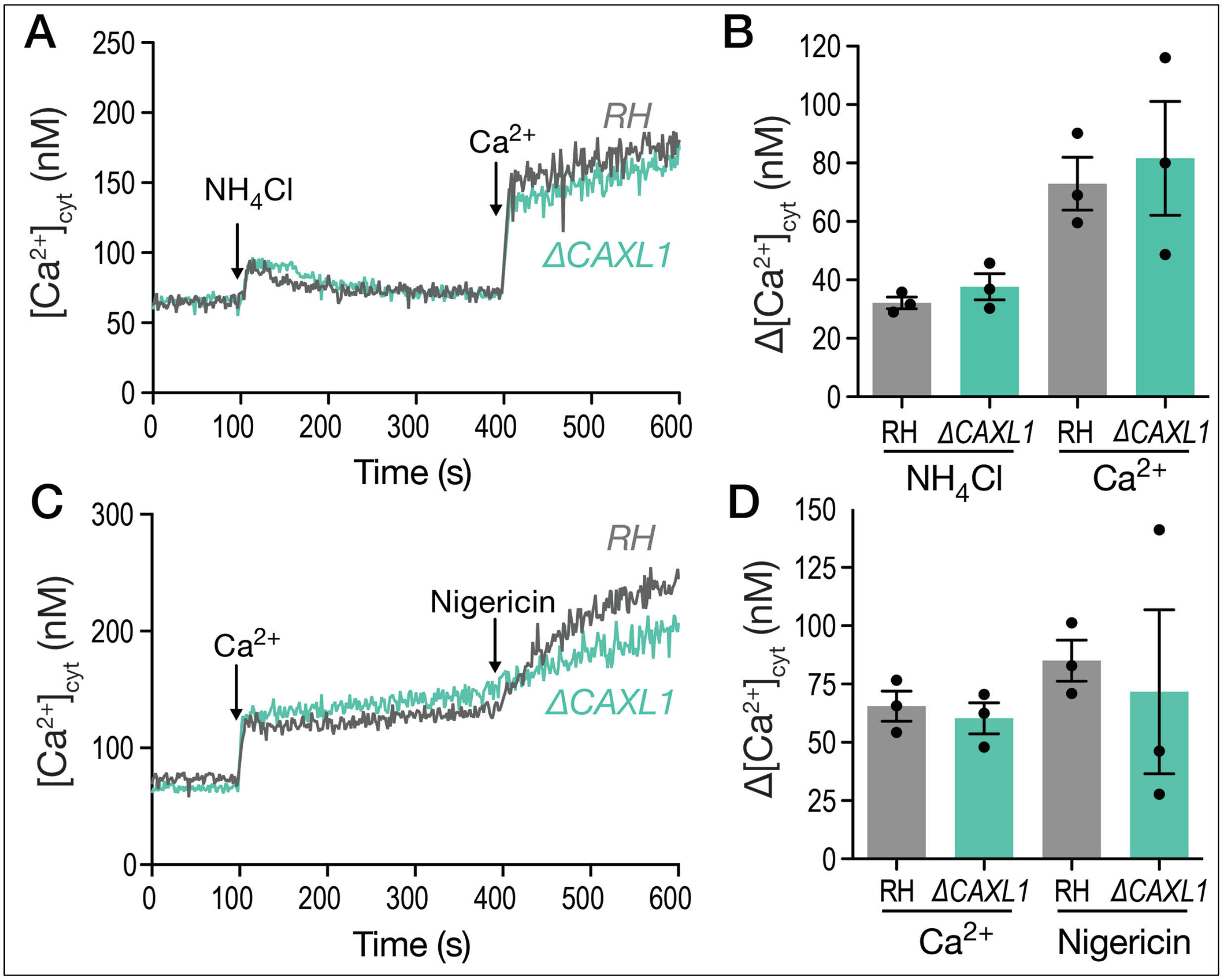

Figure S5. Acidic stores and the role of TgCAXL1. A) Cytosolic calcium concentration of tachyzoites loaded with Fura 2 and resuspended in Ringer's buffer in the presence of 100 µM EGTA. 20 mM NH_4Cl was added at 100 sec and 1.8 mM Ca^{2+} was added at 400 sec. B) Quantification of change in cytosolic calcium after adding 20 mM NH_4Cl and 1.8 mM Ca^{2+} from A). C) Cytosolic calcium concentration of tachyzoites previously loaded with Fura 2 and resuspended in Ringer's buffer with 100 µM EGTA. 1.8 mM Ca^{2+} was added at 100 sec and 10 µM Nigericin at 400 sec. D) Quantification of change in cytosolic calcium after adding 1.8 mM Ca^{2+} and after adding 10 µM Nigericin from C).

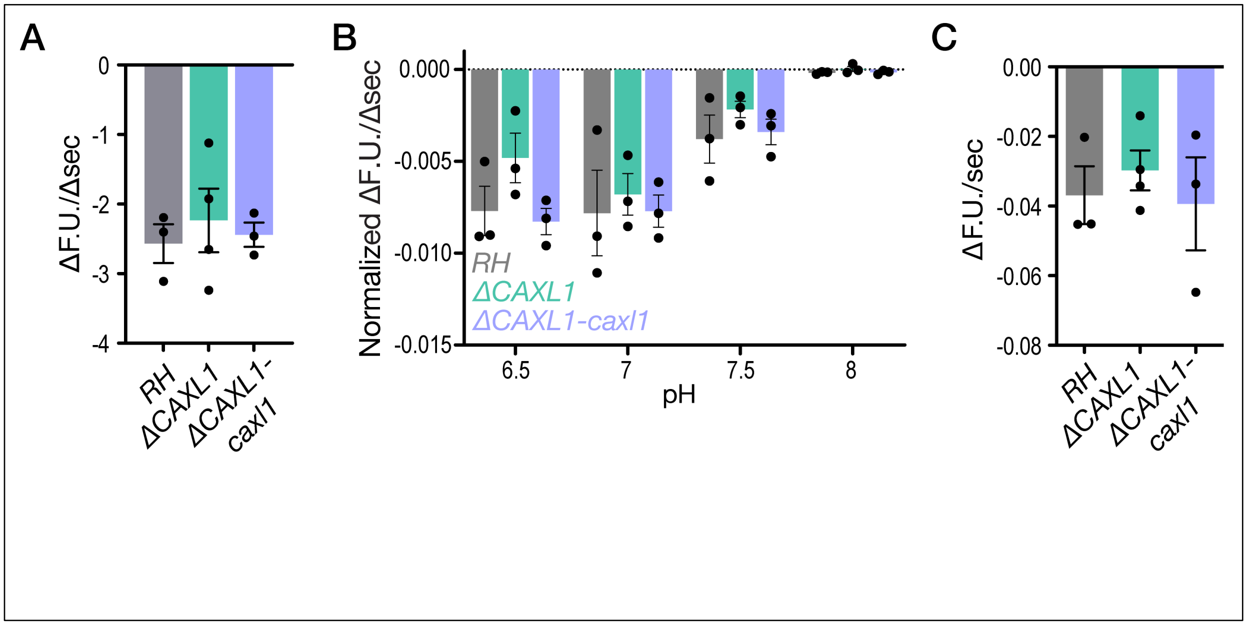

Figure S6: A) Tachyzoites of the RH (gray), *ΔCAXL1* (light green) or *ΔCAXL1-caxl1* (light blue) lines loaded with MagFluo4 were used to evaluate the rate of Ca^{2+} release after addition of A) 1 µM of ionomycin at pH 7. The data refer to Fig. 6E. Quantification of the slope after addition of 1 µM IO in parasites resuspended in CLM buffer pH 7 between 400-410 sec. B) The analysis is from the experiment shown in Fig. 6 at various pHs. Quantification of the slope after addition of 1.5 µM TG in parasites resuspended in CLM buffers with varying pHs. The slope was calculated between 400-600 sec. C) The analysis refers to data from Fig. 6B where parasites are resuspended in CLM buffer pH 7. Quantification is of the slope after addition of 1.5 µM TG from 400-440 sec.

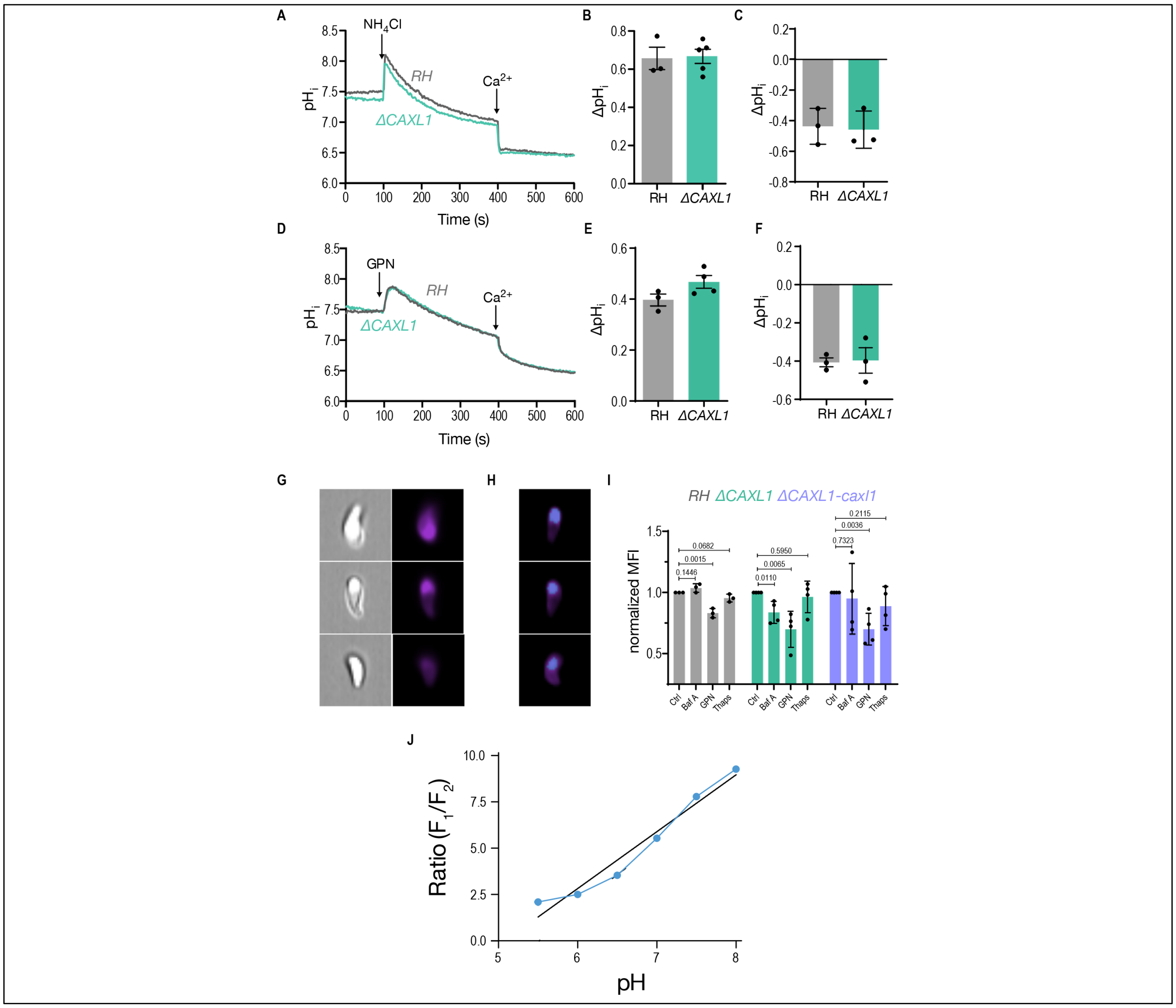

Figure S7. TgCAXL1 and intracellular pH. A) Intracellular pH levels in RH, Δ*CAXL1* and Δ*CAXL1-caxl1* cell lines loaded with BCECF-AM. Experiments were done in calcium free Standard buffer with 100 µM EGTA. At 100 sec, 20 mM NH_4Cl was added followed by 1.8 mM of Ca^{2+} at 400 sec. B) Quantification of change in pH after addition of 20 mM NH_4Cl from A. C) Quantification of change in pH after addition of Ca^{2+} from A. D) Intracellular pH levels in RH, Δ*CAXL1* and Δ*CAXL1-caxl1* cell lines loaded with BCECF. Experiments were done in calcium free Standard buffer with 100 µM EGTA. At 100s, 40 µM GPN was added followed by 1.8 mM of Ca^{2+} at 400 sec. E) Quantification of change in pH after addition of 40 µM GPN from D. F) Quantification of change in pH after addition of Ca^{2+} from D. G) The pH of acidic stores was detected using Lysosensor DND 189 in RH, Δ*CAXL1* and Δ*CAXL1-caxl1* cell lines. Representative image of parasites loaded with Lysosensor. H) Representative image of parasites loaded with Lysosensor and the blue is the fluorescence of the area measured for analysis. I) Values indicate the average of the median fluorescence intensity (MFI) from a minimum of 4-5 biological replicates. For each trial, the fluorescence of a minimum of 100 parasites for each cell line was analyzed. For BCECF experiments, at least three independent biological replicates were done for all experiments, and each experiment was done in duplicate. For quantification of ΔpH_i and MFI, mean values ± SEM were analyzed using one-way ANOVA.

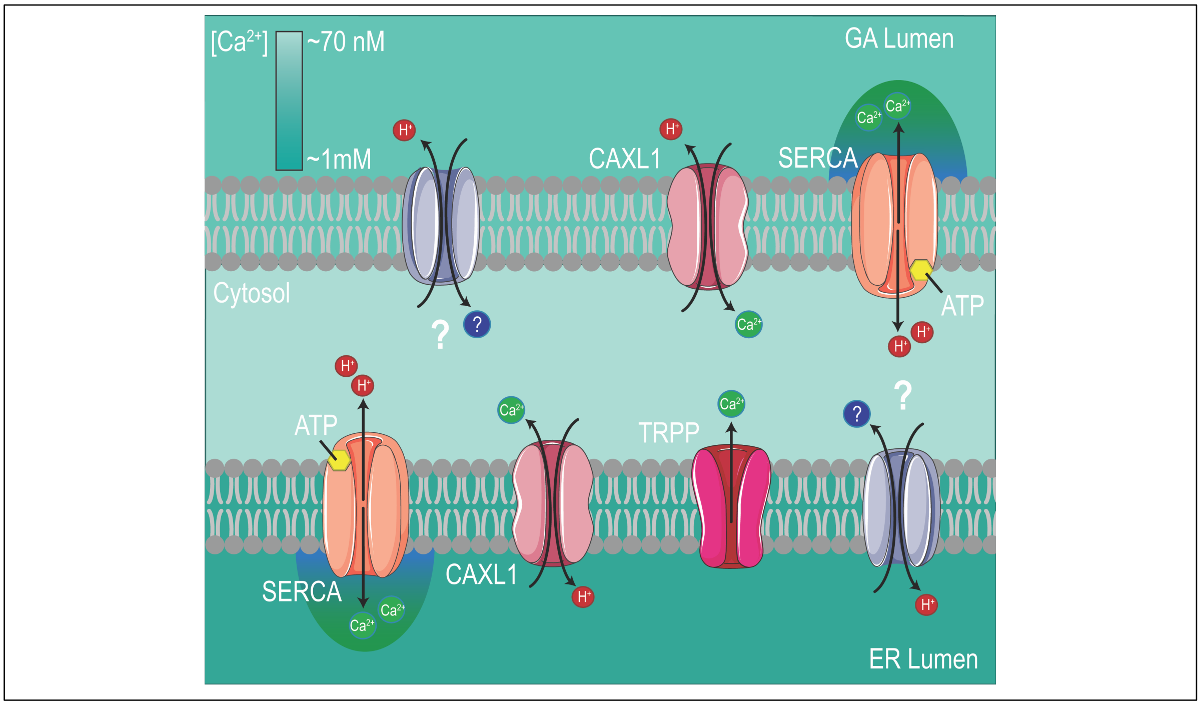

Figure S8: Intracellular calcium signaling molecules on the endoplasmic reticulum (ER) and Golgi apparatus (GA) membrane. CAXL1 is a calcium/proton exchanger expressed in the ER and GA membrane and impacts SERCA pumping of calcium into the stores. CAXL1 likely transports calcium into the cytosol due to the large calcium gradient between the stores (~500 nM-1 mM) and the cytosol (70-100 nM), in exchange for protons. For every 2 calcium ions SERCA transports into the lumen, it will transport two/three protons into the cytosol. Thapsigargin inhibits SERCA, leading to leakage of calcium into the cytosol, potentially through the TRPP channel. Finally, CAXL1 is not an essential gene, indicating that there must be other mechanisms of cation/proton exchange on the membrane of these stores, potentially another calcium or cation/proton exchanger.
